# Intra– and inter–hemispheric network dynamics supporting object recognition and speech production

**DOI:** 10.1016/j.neuroimage.2023.119954

**Published:** 2023-02-23

**Authors:** Yu Kitazawa, Masaki Sonoda, Kazuki Sakakura, Takumi Mitsuhashi, Ethan Firestone, Riyo Ueda, Toshimune Kambara, Hirotaka Iwaki, Aimee F. Luat, Neena I. Marupudi, Sandeep Sood, Eishi Asano

**Affiliations:** a Department of Pediatrics, Children’s Hospital of Michigan, Wayne State University, Detroit, 48201, USA; b Department of Neurology, Children’s Hospital of Michigan, Wayne State University, Detroit, 48201, USA; c Department of Neurosurgery, Children’s Hospital of Michigan, Wayne State University, Detroit, 48201, USA; d Department of Physiology, Wayne State University, Detroit, 48201, USA; e Department of Neurology and Stroke Medicine, Yokohama City University, Yokohama, 2360004, Japan; f Department of Neurosurgery, Yokohama City University, Yokohama, 2360004, Japan; g Department of Neurosurgery, University of Tsukuba, Tsukuba, 3058575, Japan; h Department of Neurosurgery, Juntendo University, Tokyo, 1138421, Japan; i Department of Psychology, Hiroshima University, Hiroshima, 7398524, Japan; j Department of Psychiatry, Hachinohe City Hospital, Hachinohe, 0318555, Japan; k Department of Pediatrics, Central Michigan University, Mount Pleasant, 48858, USA

**Keywords:** subdural electrocorticography (ECoG), Event-related high-gamma synchronization, Normative dynamic tractography atlas, Functional language mapping, pediatric epilepsy surgery

## Abstract

We built normative brain atlases that animate millisecond-scale intra- and inter-hemispheric white matter-level connectivity dynamics supporting object recognition and speech production. We quantified electrocorticographic modulations during three naming tasks using event-related high-gamma activity from 1,114 nonepileptogenic intracranial electrodes (i.e., non-lesional areas unaffected by epileptiform discharges). Using this electrocorticography data, we visualized functional connectivity modulations defined as significant naming-related high-gamma modulations occurring simultaneously at two sites connected by direct white matter streamlines on diffusion-weighted imaging tractography. Immediately after stimulus onset, intra- and inter-hemispheric functional connectivity enhancements were confined mainly across modality-specific perceptual regions. During response preparation, left intra-hemispheric connectivity enhancements propagated in a posterior-to-anterior direction, involving the left precentral and prefrontal areas. After overt response onset, inter- and intra-hemispheric connectivity enhancements mainly encompassed precentral, postcentral, and superior-temporal (STG) gyri. We found task-specific connectivity enhancements during response preparation as follows. Picture naming enhanced activity along the left arcuate fasciculus between the inferior-temporal and precentral/posterior inferior-frontal (pIFG) gyri. Nonspeech environmental sound naming augmented functional connectivity via the left inferior longitudinal and fronto-occipital fasciculi between the medial-occipital and STG/pIFG. Auditory descriptive naming task enhanced usage of the left frontal U-fibers, involving the middle-frontal gyrus. Taken together, the commonly observed network enhancements include inter-hemispheric connectivity optimizing perceptual processing exerted in each hemisphere, left intra-hemispheric connectivity supporting semantic and lexical processing, and inter-hemispheric connectivity for symmetric oral movements during overt speech. Our atlases improve the currently available models of object recognition and speech production by adding neural dynamics via direct intra- and inter-hemispheric white matter tracts.

## 1. Introduction

Humans begin to name objects based on their visual appearance and environmental sounds during toddlerhood, and children subsequently acquire the ability to understand and communicate with spoken sentences by preschool (Bloom, 1968; Pinker, 2009). Older children and adults recognize a bird based on its characteristic wings, chirping sounds, or a spoken question like “*What flies in the sky*?”. Investigators suggest object recognition and speech production result from coordinated interactions of multiple cortical regions (Duffau, 2008; Lorca-Puls et al., 2021). The collective evidence from lesion-deficit studies of stroke survivors, electrical stimulation mapping of patients undergoing brain surgery, and functional MRI (fMRI) studies of healthy and diseased individuals has successfully identified the anatomical networks supporting object recognition and speech production. The stimuli presented in these studies included visual images (Laird et al., 2005; DeLeon et al., 2007; Baldo et al., 2013; Hamberger et al., 2014; Tate et al., 2014; Gajardo-Vidal et al., 2021; Lu et al., 2021), non-speech environmental sounds (Tanaka et al., 2002; Saygin et al., 2003; Lewis et al., 2004; Engel et al., 2009; Schirmer et al., 2012), and spoken questions (Saygin et al., 2003; Vigneau et al., 2006; Schirmer et al., 2012; Hamberger et al., 2014; Nakai et al., 2017). The causal significance of intra-hemispheric white matter networks in speech production was further clarified in a study of 134 stroke survivors, which revealed damage to the left arcuate fasciculus originating from the left prefrontal cortices was more predictive of permanent speech impairment compared with damage restricted to the overlying prefrontal cortices (Gajardo-Vidal et al., 2021). Inter-hemispheric white matter networks likewise play a causal role in object recognition by transferring necessary information mainly via the corpus callosum; this notion derives from the observation that many patients with drug-resistant epilepsy develop naming impairment following corpus callosotomy (Lausberg et al., 2003; Bloom and Hynd, 2005; Gazzaniga, 2005). However, the exact spatio-temporal dynamics of inter-hemispheric neural interactions during object recognition remain unknown. Understanding of the inter-hemispheric connectivity dynamics is expected to improve the currently available models of object recognition and speech production (Hickok and Poeppel, 2007; Rauschecker and Scott, 2009; Dick et al., 2014; Chang et al., 2015; Skeide and Friederici, 2016) and the prediction of cognitive outcomes following white matter damage.

The present study aimed to build a normative brain atlas that visualizes the millisecond-scale dynamics of naming task-related modulations of functional connectivity via specific white matter pathways. We assessed event-related modulations of high-gamma activity (70–110 Hz) on intracranial EEG (iEEG) recording (Sinai et al., 2005; Towle et al., 2008; Lachaux et al., 2012) in relation to the anatomical white matter tracts (Yeh et al., 2018) delineated by diffusion-weighted imaging (DWI) tractography (Catani et al., 2005; Duffau, 2008; Dick and Tremblay, 2012; David et al., 2013; Sonoda et al., 2021; Yamao et al., 2021). High-gamma augmentation and attenuation are surrogate markers of cortical activation and deactivation, respectively. For example, high-gamma amplitude (i.e., square root of iEEG band power) is well correlated with neural firing rate (Mukamel et al., 2005; Ray et al., 2008; Leszczyński et al., 2020), hemodynamic responses (Harvey et al., 2013; Kunii et al., 2013b; Hill et al., 2021), and glucose metabolism (Nishida et al., 2008). The strength of iEEG-based brain mapping over fMRI is its excellent temporal resolution, and that over non-invasive EEG recording includes its resilience to artifacts derived from overt vocalization (Ball et al., 2009). Previous iEEG studies of patients with focal epilepsy visualized the rapid dynamics of cortical modulations that support picture naming (Edwards et al., 2010; Kunii et al., 2013a; Abel et al., 2015; Collard et al., 2016; Dubarry et al., 2017; Riès et al., 2017; Giahi Saravani et al., 2019; Nakai et al., 2019; Ervin et al., 2021) and spoken questions (Cervenka et al., 2013; Nakai et al., 2017; Forseth et al., 2018). However, the dynamics of object recognition based on nonspeech environmental sounds have remained under-studied. Studying this area is expected to clarify the spatiotemporal dynamics of brain connectivity modulations unrelated to syntactic processing.

The present study built on the notion that spatially distinct, yet concurrent high-frequency neural events generated by cortico-cortical networks underly sensorimotor and cognitive processes (Singer, 1993, 2018; Buonomano and Merzenich, 1998). Previous iEEG studies have suggested that linguistic processes are supported by networks of widespread discrete cortical regions based on group-level observations that naming task-related high-gamma augmentations at remote brain regions temporally overlapped or time-locked (Leuthardt et al., 2012; Collard et al., 2016; Riès et al., 2017; Arya et al., 2019; Giahi Saravani et al., 2019). Furthermore, our recent iEEG study demonstrated that cortical sites simultaneously showing task-related high-gamma augmentation were associated with increased effective connectivity rated by spectral responses to single-pulse electrical stimulation (Sonoda et al., 2021). Compared to lower-frequency bands, event-related high-gamma activity has a finer temporal resolution and a higher value in predicting language outcomes following resective surgery (Sonoda et al., 2022). Thus, the present study quantified naming-related functional connectivity based on cortical areas linked via direct DWI streamlines that displayed significant, simultaneous, and sustained task-related high-gamma modulations. This analytic approach is conceptually similar to fMRI-based studies of group-level functional connectivity, in which distinct cortical regions showing statistically similar task-related hemodynamic activation patterns were considered functionally connected (Sun et al., 2004; Crossley et al., 2013; Glasser et al., 2016). The methodological novelty of the present iEEG/DWI study is an animation of rapid modulations of *naming task-related* functional connectivity via distinct *intra-* and *inter-hemispheric* white matter pathways. Including DWI-based anatomical tracts is expected to enhance the biological plausibility of functional connectivity suggested by iEEG analysis. We generated video atlases to clarify the network dynamics which support object recognition based on images, nonspeech environmental sounds, and spoken questions.

The current study determined intra- and inter-hemispheric connectivity enhancement patterns after stimulus onset, before response onset, and after response onset. We specifically hypothesized that inter-hemispheric functional connectivity across modality-specific perceptual areas would be enhanced within 300 ms after stimulus onset (i.e., the period when perceptual processes should have already taken place). This hypothesis was based on previous observations of patients who, following corpus callosotomy, developed pronounced impairment in naming objects explicitly presented to the left visual field; investigators attributed such visual field-specific naming impairment to failed inter-hemispheric transfer of mental representations of perceptual inputs (Lausberg et al., 2003; Bloom and Hynd, 2005; Gazzaniga, 2005). Speech impairment following callosotomy was found to be more severe when disconnection involved the posterior callosum in addition to the anterior two-thirds (Kawai et al., 2004). Thus, we hypothesized that the posterior corpus callosum would support naming task-related enhancement of inter-hemispheric functional connectivity. We expected that inter-hemispheric connectivity enhancements would take place within 300 ms after stimulus onset because a study of 16 healthy individuals using magnetoencephalography (MEG) showed that inter-hemispheric alpha-band coherence was grossly increased within 300 ms after visual presentation of a word stimulus to the left visual field (Doron et al., 2012). We also hypothesized that inter-hemispheric functional connectivity between left and right sensorimotor areas would be enhanced after response onset. This hypothesis is primarily based on the notion that speech production requires symmetric and synchronous oral and vocal movements. In addition, we expected that left intra-hemispheric connectivity involving the left prefrontal and temporal lobe cortices would be enhanced during response preparation, as proposed in many neurobiological models of speech production (Hickok and Poeppel, 2007; Glasser and Rilling, 2008; Ellmore et al., 2009; Rauschecker and Scott, 2009; Catani et al., 2012; Dick et al., 2014; Gierhan, 2013; Han et al., 2013; Chang et al., 2015; Skeide and Friederici, 2016; Gajardo-Vidal et al., 2021).

In the present study, we compared the dynamics of intra- and inter-hemispheric cortical and white matter connectivity modulations between picture and nonspeech environmental sound naming tasks. These naming tasks require domain-specific - visual versus auditory - perceptual processes followed by semantic recognition, lexical retrieval, overt vocalization, and monitoring of vocal responses (Glaser, 1992; Lewis et al., 2004; Houix et al., 2012). Comparing the network dynamics between the picture and nonspeech environmental sound naming tasks is expected to clarify the spatiotemporal profiles of modality-specific intra- and inter-hemispheric functional connectivity modulations. Healthy individuals can recognize a brief (e.g., 120 ms), static visual stimulus; in contrast, sound-based object recognition requires prolonged (e.g., 2 s) analysis of non-stationary auditory stimuli, more extensive storage and manipulation of mental representations, and subsequent lexical selection (Grill-Spector et al., 2000; Chu et al., 2009). Response preparation processes, including lexical retrieval, are suggested to occur for several hundred ms before overt vocalization (Sonoda et al., 2021). Thus, we hypothesized that nonspeech environmental sound-based naming, compared to picture naming, would require more sustained or extensive functional connectivity enhancements across the left prefrontal region, before an overt response.

Delineating the differences in network dynamics between environmental sound and auditory descriptive naming tasks is expected to increase understanding of the textual/syntactic process. In this comparison, recognition of textual/syntactic features is required in descriptive naming alone, while the stimulus modality was commonly auditory. We hypothesized that spoken questions, compared to environmental sounds, would more extensively enhance functional connectivity across the left prefrontal regions before an overt response. This hypothesis was driven by the observation that electrical stimulation of left posterior frontal lobe sites interfered with syntactic encoding, without impairing overt picture naming (Chang et al., 2018). fMRI studies also commonly suggest that syntactic processing is supported by large-scale intra-hemispheric connectivity involving the left prefrontal regions (Fedorenko et al., 2012; Hagoort and Indefrey, 2014; Chen et al., 2021).

## 2. Material and methods

### 2.1. Participants

The inclusion criteria were [a] patients with drug-resistant focal epilepsy, [b] extraoperative iEEG recording as part of the epilepsy presurgical evaluation at Children’s Hospital of Michigan, Detroit Medical Center between January 2015 and December 2018, [c] ability to complete naming tasks based on pictures, nonspeech environmental sounds, and spoken questions during the iEEG recording, and [d] aged five years or older. The exclusion criteria were [i] extensive brain malformations deforming the central, lateral, or calcarine sulci, [ii] previous resective epilepsy surgery, [iii] a visual field deficit on confrontation, [iv] historical hearing deficits, [v] inability to perform naming tasks due to severe developmental delays, and [vi] right-hemispheric language dominance as estimated by left-handedness associated with left-hemispheric congenital neocortical lesions (Rasmussen and Milner, 1977; Akanuma et al., 2003; Möddel et al., 2009). We extensively discussed the rationale and validity for estimating the language-dominant hemisphere based on handedness and anatomical imaging (Sonoda et al., 2022). Thus, we analyzed 13 right-handed children who satisfied the eligibility criteria (age: 6–19 years; Table 1). The current study was approved by the Wayne State University Institutional Review Board. Written informed consent was obtained from each patient’s parents or legal guardians, and assent was obtained from patients older than 13 years.

### 2.2. Intracranial electrode placement and extraoperative iEEG recording

Intracranial EEG data acquisition was conducted as described in our previous studies (Nakai et al., 2017; Kambara et al., 2018; Sonoda et al., 2022). An experienced neurosurgeon implanted platinum disk electrodes (center-to-center distance: 10 mm) on the pia mater of the affected hemisphere to localize the boundaries between the seizure onset zone (Asano et al., 2009) and eloquent areas (Nakai et al., 2017; Sonoda et al., 2022). At the inpatient ward, each patient underwent continuous video-iEEG recording for 3 to 7 days with a sampling rate of 1,000 Hz using a Neurofax Digital EEG system (Nihon Kohden America Inc., Foothill Ranch, CA, USA). To minimize the unwanted effects of epileptiform discharges on the measurement of task-related high-gamma amplitude modulations (Mercier et al., 2022), we only included nonepileptic electrode sites (i.e., those not affected by the seizure onset zone, interictal spike discharges, or MRI-based structural lesions) for further iEEG analysis. Thus, we included 1114 artifact-free nonepileptic electrode sites (57–112 per patient) and assessed iEEG data using a common average reference (Sonoda et al., 2022).

### 2.3. MRI data analysis

**Fig. 1.**
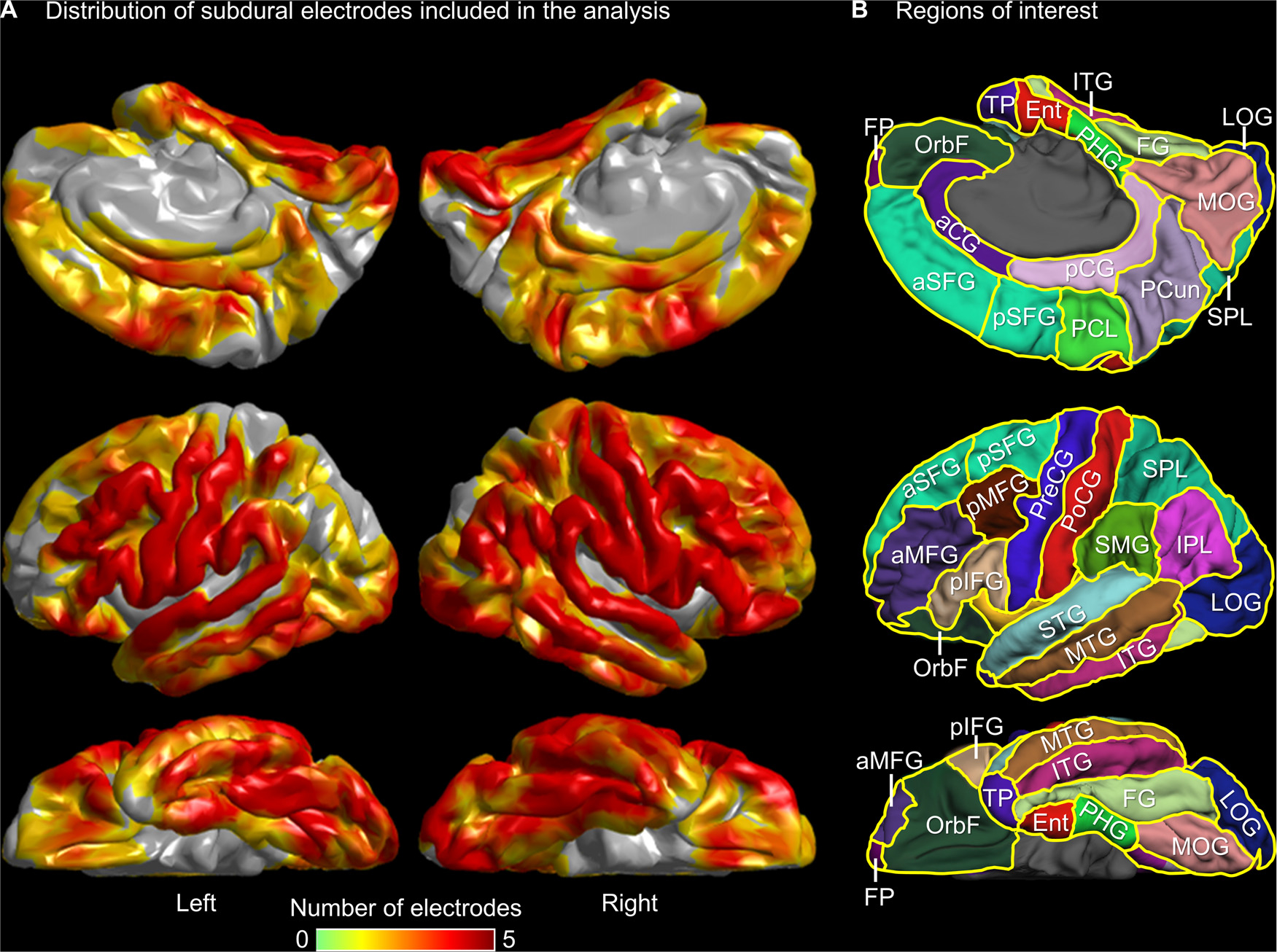
Regions of interest (ROIs) and distribution of subdural electrode sites included in the analysis. (A) The pooled distribution of artifact-free, nonepileptic electrode sites from *n* = 13 patients. (B) ROI locations on the left hemisphere are presented. PreCG: precentral gyrus. PoCG: postcentral gyrus. STG: superior-temporal gyrus. aMFG and pMFG: anterior and posterior middle-frontal gyrus. SMG: supramarginal gyrus. aSFG and pSFG: anterior and posterior superior-frontal gyrus. FG: fusiform gyrus. pIFG: posterior inferior-frontal gyrus (summation of pars opercularis and pars triangularis). MTG: middle-temporal gyrus. LOG: lateral occipital gyrus. ITG: inferior-temporal gyrus. OrbF: orbitofrontal gyrus (summation of pars orbitalis and medial and lateral orbitofrontal gyri). MOG: medial occipital gyrus (summation of cuneus and lingual gyri). SPL: superior parietal lobule. PCun: Precuneus gyrus. pCG: posterior cingulate gyrus. A total of 36 regions mentioned above (18 on each hemisphere) were included in the ROI-based analysis. The following electrode sites were not included in the group-level region of interest (ROI) analysis because the number of electrode sites was below nine (see below). aCG: anterior cingulate gyrus. PCL: paracentral lobule. FP: frontal pole. IPL: inferior parietal lobule. TP: temporal pole. Ent: entorhinal gyrus. PHG: parahippocampal gyrus. Table S1 provides the exact number of artifact-free, nonepileptic electrode sites in given ROIs.

For each patient, we reconstructed the three-dimensional MRI cortical surface with all corresponding electrode sites registered directly on the cortical surface (Nakai et al., 2017; Stolk et al., 2018; Sakakura et al., 2022). We confirmed the spatial accuracy of electrode-MRI coregistration using visual assessment of intraoperative photographs (Pieters et al., 2013). FreeSurfer scripts (http://surfer.nmr.mgh.harvard.edu) then normalized the coordinates of a given electrode site into standardized ‘FSaverage’ space (Ghosh et al., 2010; Nakai et al., 2017). The script automatically parcellated the cortical gyri and assigned each electrode site a corresponding region of interest (ROI) based on Desikan’s atlas (Fig. 1; Desikan et al., 2006; Nakai et al., 2017; Ono et al., 2022).

### 2.4. Naming tasks

**Fig. 2.**
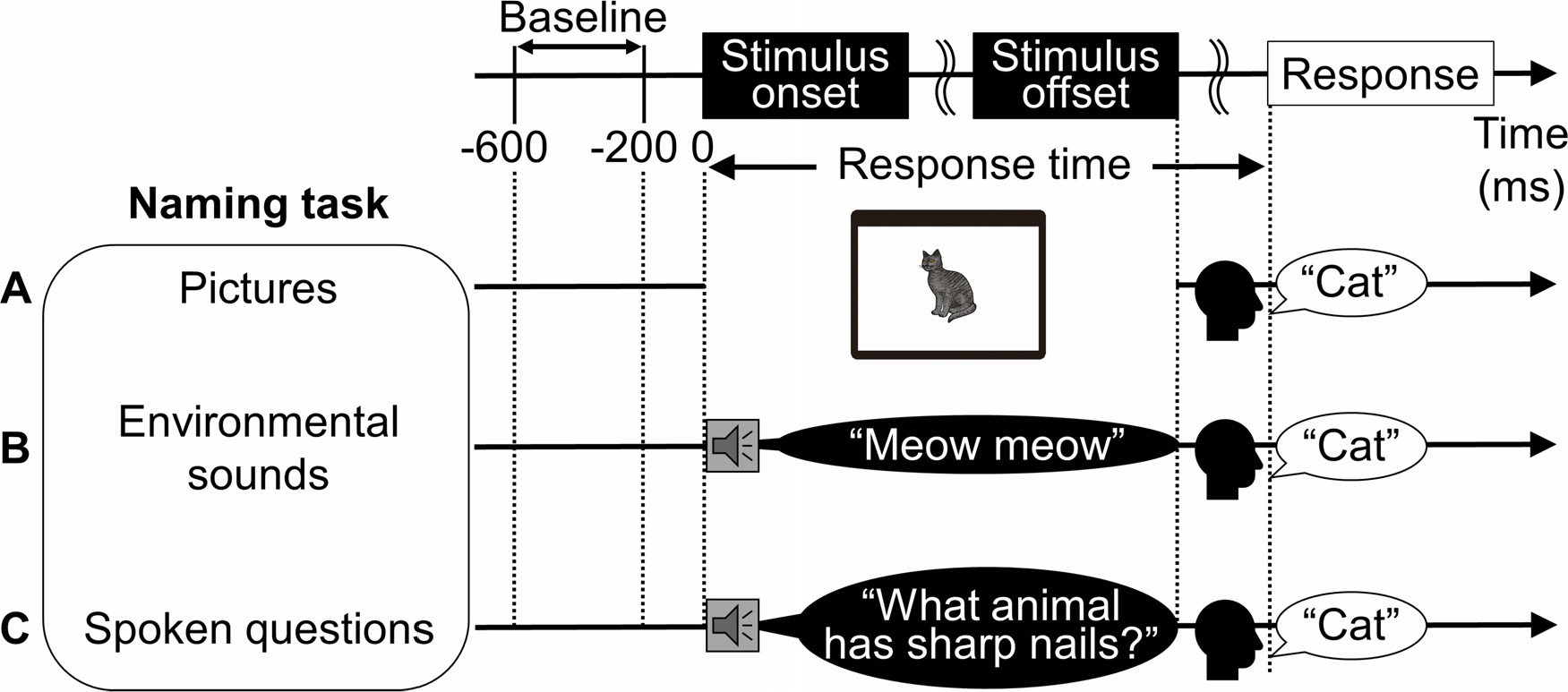
Naming tasks. (A) Picture naming. (B) Nonspeech environmental sound naming. (C) Auditory descriptive naming. The baseline/reference period was the 400-ms between 200 and 600 ms prior to the stimulus onset, and the response time was defined as the period between stimulus onset and response onset. Note that some patients responded before stimulus offset in picture and nonspeech environmental sound naming. In contrast, all patients responded after stimulus offset in auditory descriptive naming.

We employed simple and patient-friendly tasks to localize the eloquent networks as routine epilepsy presurgical evaluation (Kambara et al., 2018; Sonoda et al., 2022). During interictal extraoperative iEEG recording, each patient underwent the following three tasks requiring overt naming based on either [a] pictures, [b] nonspeech environmental sounds, or [c] spoken questions (Fig. 2). Patients were instructed to say ‘I don’t know’ if they did not know the correct answer. An investigator pressed the stimulus presentation button immediately following each patient’s response, to initiate the next stimulus 2 or 2.5 s later; consequently, the inter-trial intervals varied depending on how long each participant took for a given response. The response time was defined as the period between stimulus onset and response onset (Fig. 2).

#### 2.4.1. Picture naming task

In each of the 60 trials, patients named the drawing of a common object, ranging from 5.1 to 6.1 cm in width and height (i.e., 4.9° to 5.8°), presented for 1.8 s on the center of a 19-inch LCD monitor placed 60 cm in front of the patients.

#### 2.4.2. Nonspeech environmental sound naming task

In each of the 60 trials, patients named an environmental sound stimulus (e.g., *cat, train, guitar, phone, and thunder*) presented for 1.8 s with an approximate intensity of 70 dB, via two open-field speakers.

#### 2.4.3. Auditory descriptive naming task

In each of the 100 trials, patients named an object relevant to a given spoken question lasting 1.8 s (e.g., *What flies in the sky?*) and presented with an approximate intensity of 70 dB, via two open-field speakers. A given question began with either *What, Who, When*, or *Where*. Stimulus presentations, patient responses, and iEEG signals were synchronized using an optical sensor and microphone (Kambara et al., 2018).

### 2.5. iEEG analysis

#### 2.5.1. Time–frequency analysis

We quantified the degree of task-related high-gamma modulations at given electrode sites using the methods reported previously (Brown et al., 2008; Nakai et al., 2017, 2019). The complex demodulation algorithm implemented in BESA Analysis Software (BESA GmbH, Gräfelfing, Germany; Papp and Ktonas, 1977; Hoechstetter et al., 2004) multiplied each iEEG time-voltage signal with a complex exponential followed by a low pass filter, in steps of 10 ms and 5 Hz. It was a finite impulse response filter of Gaussian shape; thus, the complex demodulation used here was effectively equivalent to a Gabor transformation. The filter mentioned above had a full width at half maximum of 15.8 × 2 ms in the temporal and 7.1 × 2 Hz in the frequency domain.

The analysis periods of interest included: [1] a 1100-ms period around stimulus onset (i.e., 200-ms pre- and 900-ms post-stimulus onset), [2] a 1800-ms period centered on the stimulus offset, and [3] a 1800-ms period centered on the response onset, and the total number of 10-ms time bins was 473. At each 10-ms bin, we determined the percent change of high-gamma amplitude (a measure proportional to the square root of power), at 70–110 Hz, relative to the baseline mean between 600 and 200 ms before stimulus onset. We excluded trials without correct answers and those without quiescence during the baseline period (Nakai et al., 2019).

#### 2.5.2. Visualization of cortical high–gamma modulations

**Fig. 3.**
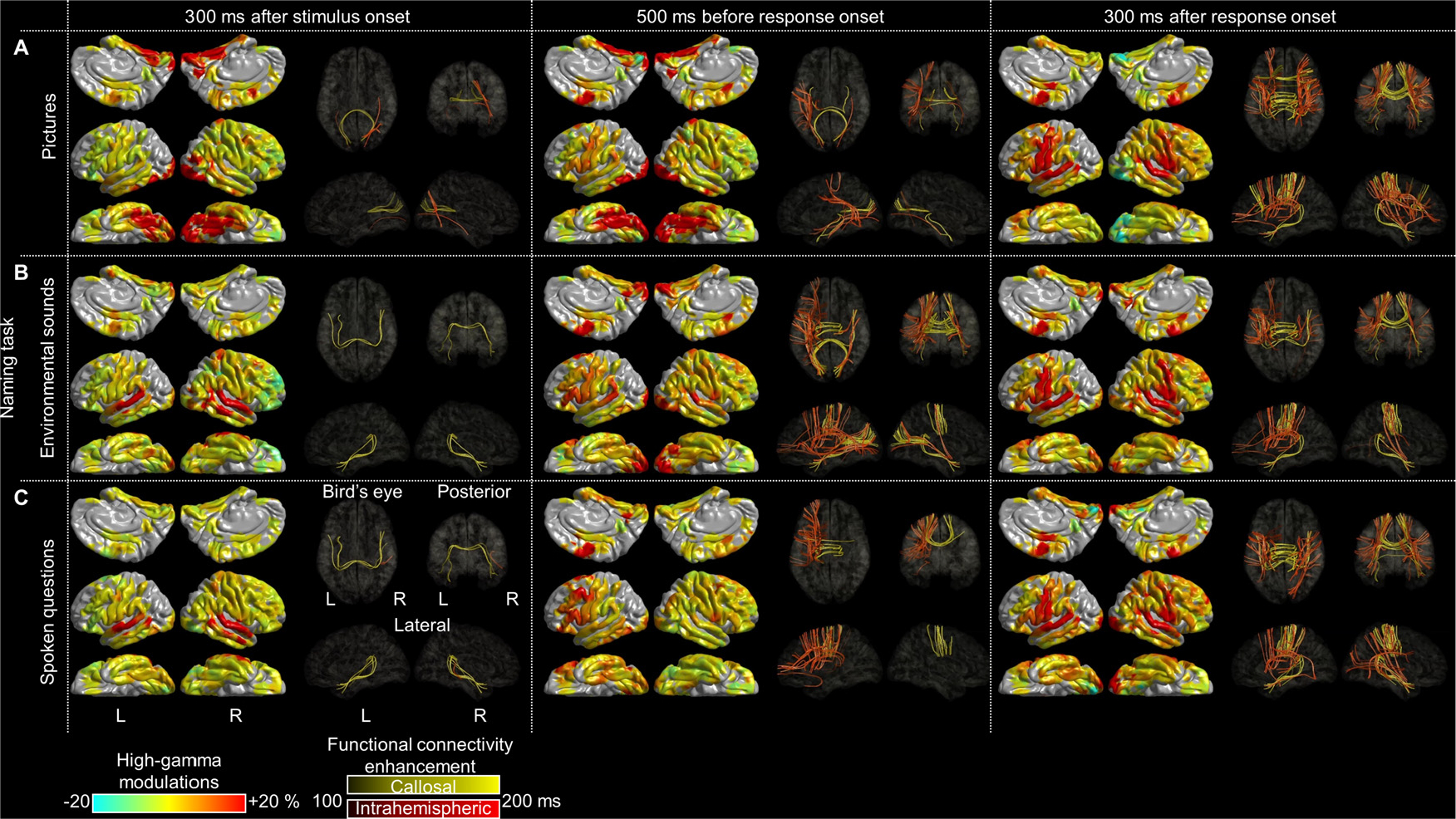
Snapshots of task-related cortical and functional connectivity modulations. (A) Picture naming (Video S1). (B) Nonspeech environmental sound naming (Video S2). (C) Auditory descriptive naming (Video S3). Left column: 300 ms after stimulus onset. Middle column: 500 ms before response onset. Right column: 300 ms after response onset. Cortical surface images show the percent change of cortical high-gamma modulations compared to the baseline period between 200 and 600 ms before stimulus onset. Dynamic tractography images present the white matter pathways with significant intra-hemispheric (red) and inter-hemispheric (yellow) functional connectivity enhancement.

We generated a group-level movie atlas with 10 ms resolution that animates cortical high-gamma amplitude modulations on a spatially-normalized, 3-dimensional brain surface (i.e., FSaverage) (Fig. 3; Videos S1–S3), as previously reported (Nakai et al., 2019). The percent change in high-gamma (70–110 Hz) amplitude at each electrode site was interpolated within 10 mm from the electrode center, and high-gamma amplitudes averaged across electrodes pooled from all patients were presented on the FreeSurfer standard brain template.

### 2.6. Statistical assessment on behavioral analysis

The Friedman test determined whether the correct response rate and the median response time of given patients differed among three naming tasks, and it was followed by a pairwise comparison using the Wilcoxon signed-rank test (SPSS Statistics 28; IBM Corp., Chicago, IL, USA). A two-sided p-value of <0.05 was considered statistically significant. We calculated the Pearson correlation coefficient *r* using the Z-value from the Wilcoxon Signed-Rank test, to assess the effect size of the between-task difference. The formula was $r=Z/\surd \left(\text{N}\right)$ where Z equals the Z-value, and N equals the study size (de Lima et al., 2021).

### 2.7. Statistical assessment on cortical high–gamma modulations

**Fig. 4.**
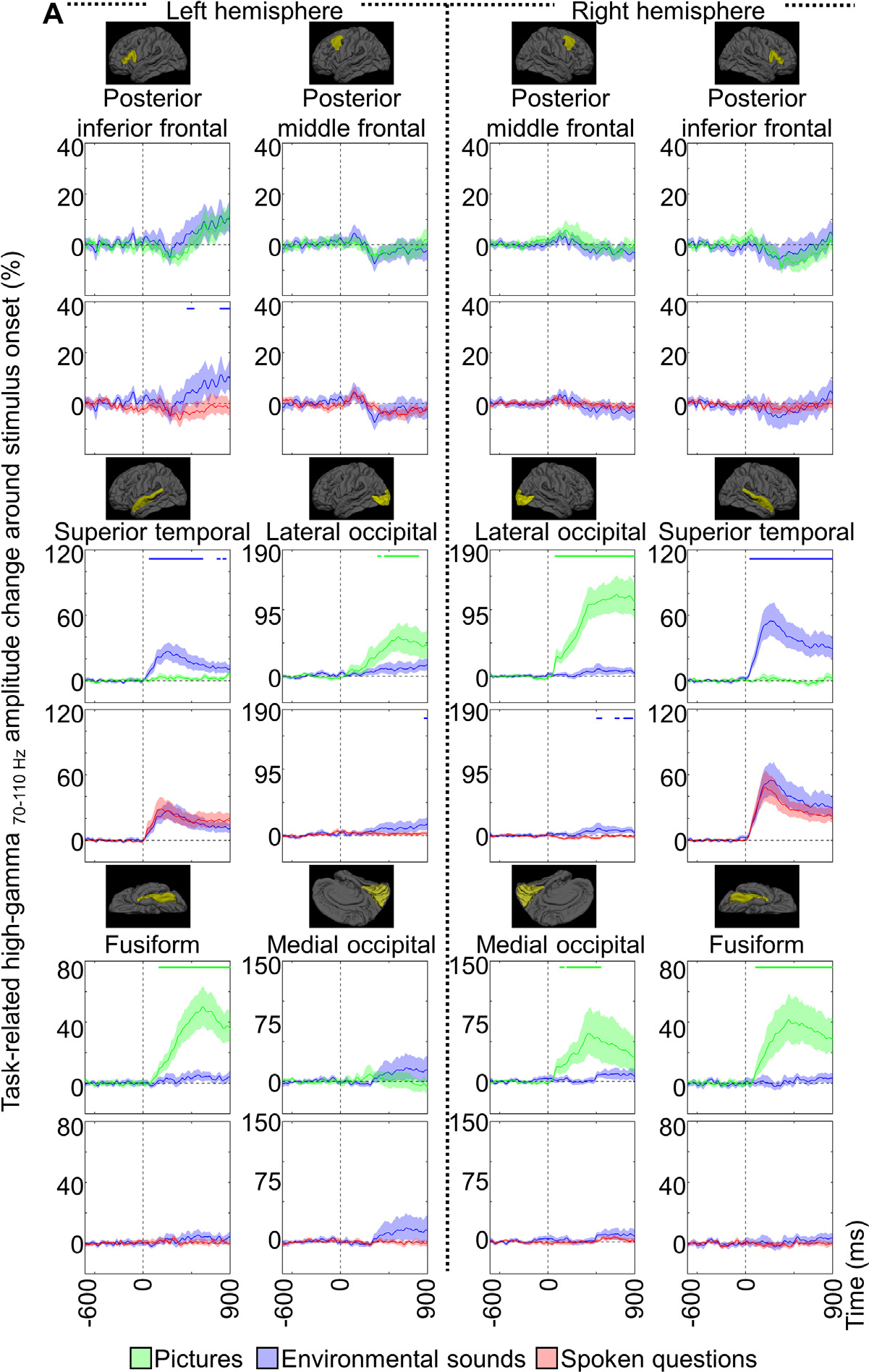

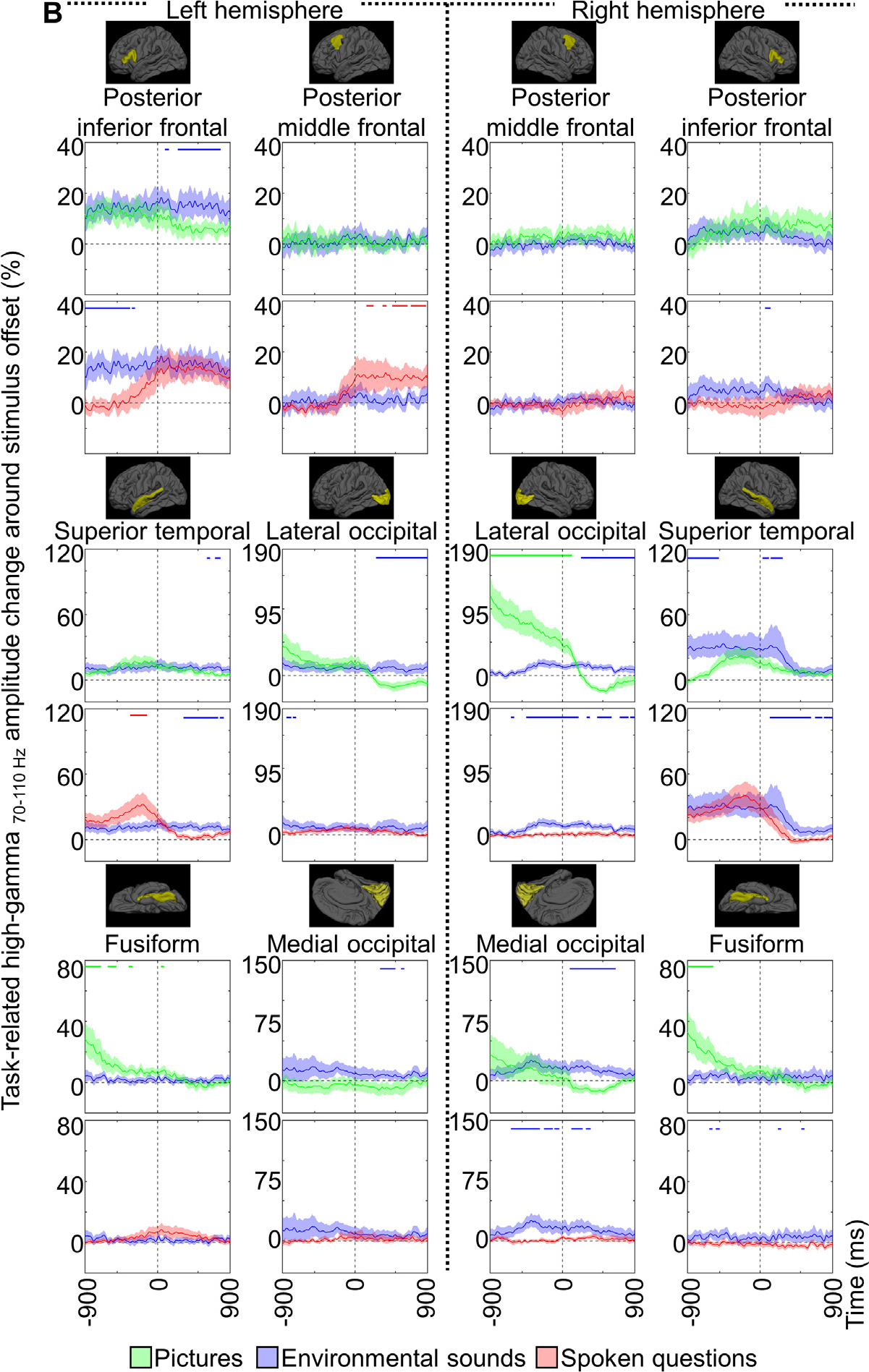

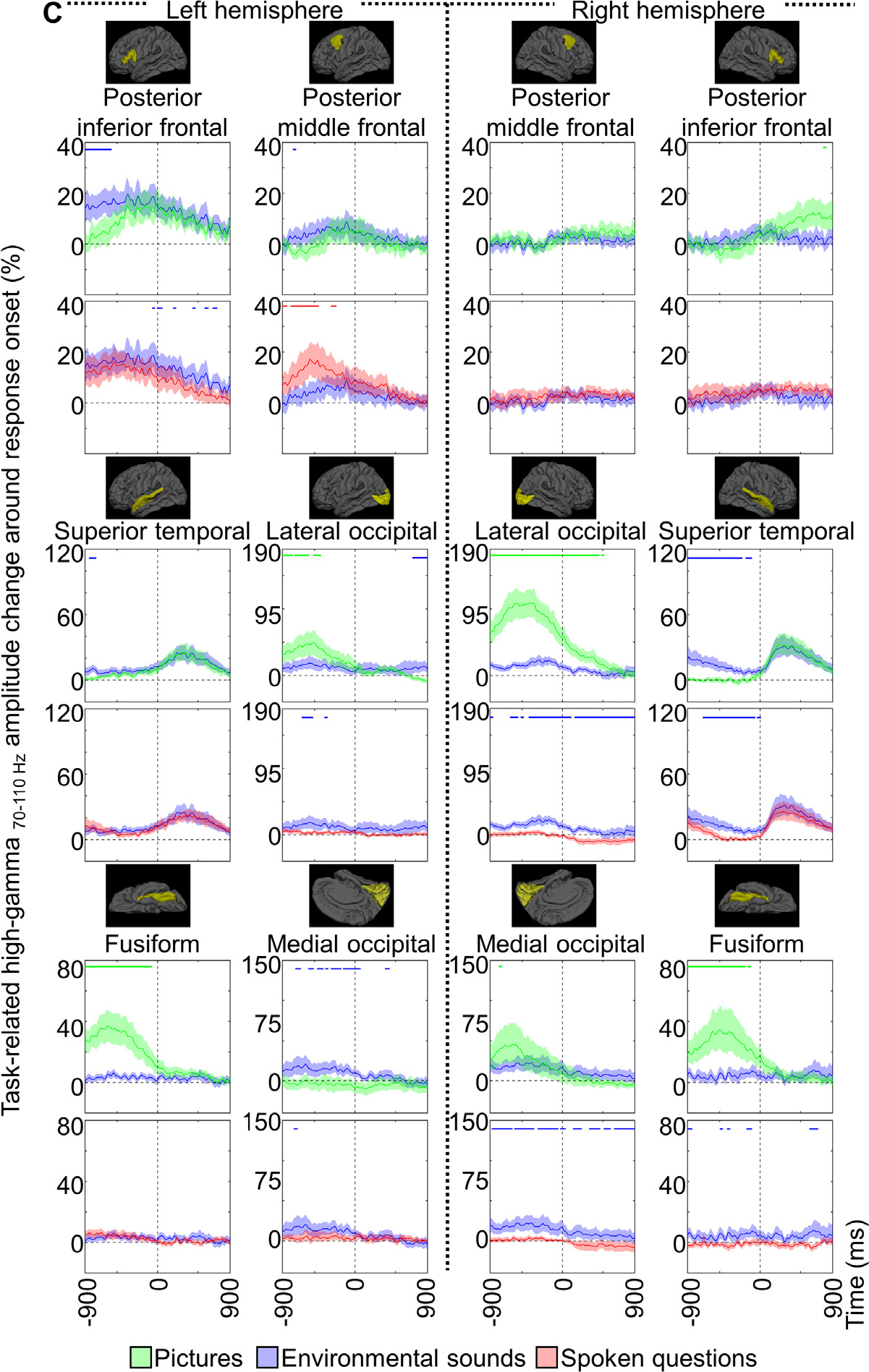
Cortical high-gamma modulations at regions of interest (ROIs). A given plot presents the percent change in high-gamma amplitudes at each anatomical ROI compared to the baseline mean at 200 to 600 ms before stimulus onset. (A) −600 to 900 ms around stimulus onset. (B) −900 to 900 ms around stimulus offset. (C) −900 to 900 ms around response onset. Light green: picture naming. Blue: nonspeech environmental sound naming. Magenta: auditory descriptive naming. Shade: 95% bootstrap confidence intervals. Horizontal bars indicate the periods in which high-gamma amplitudes were significantly greater during one task than the other based on the Studentized bootstrap statistics. In the upper row, high-gamma amplitudes during picture naming were contrasted with those during nonspeech environmental sound naming. In the lower row, those during nonspeech environmental sound naming were contrasted with auditory descriptive naming. Video S4 presents plots from all 36 ROIs.

At each ROI (Fig. 1), we plotted the mean task-related high-gamma amplitude with a 95% bootstrap confidence interval (CI) shade, across all artifact-free, nonepileptic electrode sites (Fig. 4; Video S4). We employed this analysis at all ROIs containing at least nine eligible electrode sites on each hemisphere. Table S1 provides 36 ROIs (18 per hemisphere) satisfying this criterion and included in the analysis.

Studentized Bootstrap statistics (resampled 2000 times) determined when and where (i.e., at what cortical ROIs) task-related high-gamma amplitudes differed from baseline (Nakai et al., 2019; Fig. 4; Video S4). We considered task-related high-gamma augmentation significant when the lower limit of the 99.99% CI of the mean high-gamma amplitude ranged beyond zero for ≥50-ms consecutively. We likewise considered task-related high-gamma attenuation significant when the upper limit of the 99.99% CI of the mean high-gamma amplitude ranged below zero for ≥50-ms consecutively. Employment of a 99.99% CI is equivalent to a Bonferroni correction for 500 repeated comparisons, and a consecutive 50-ms period contains at least three high-gamma oscillatory cycles. Such a strict significance threshold increased the risk of Type II error but reduced Type I error.

The studentized Bootstrap statistics determined when and where task-related high-gamma amplitudes differed between the pictures and nonspeech environmental sounds. High-gamma amplitudes at given cortical ROIs significantly differed between two tasks when the lower limit of the 99.99% CI of the mean difference in high-gamma amplitude ranged beyond zero for ≥50-ms consecutively. Video S4 presents results from the whole-brain level statistical analyses for all time windows. Likewise, we determined when and where task-related high-gamma amplitudes differed between the nonspeech environmental sounds and spoken questions (Fig. 4; Video S4).

### 2.8. Statistical assessment on white matter functional connectivity modulations

With DWI tractography data, we visualized the dynamics of direct white matter functional connectivity between cortical ROIs *simultaneously* showing significant task-related high-gamma augmentation (or attenuation), for at least 100 ms (i.e., ≥ consecutive ten 10-ms time-bins; Video S1: pictures; Video S2: nonspeech environmental sounds; Video S3: spoken questions). We considered functional connectivity between cortical ROIs to be enhanced (or reduced) only if both regions showed significant, simultaneous, and sustained cortical high-gamma augmentation (or attenuation) and if they were accompanied by direct DWI streamlines (Ono et al., 2022). If significant high-gamma augmentation was noted in χ% of the 473 time-bins (i.e., 4,700 ms analysis periods) on average across 36 cortical ROIs, the chance probability (i.e., Type I error) of high-gamma co-augmentation lasting ≥100 ms was $\approx \left(36/2\right)\times \left(36-1\right)\times \left(473-10+1\right){\left\{\left(\chi /100\right)\times \left(\chi /100\right)\right\}}^{10}$. Thereby, we treated the onset latency of high-gamma co-augmentation as the beginning of a given connectivity enhancement.

### 2.9. Visualization of white matter functional connectivity modulations

We delineated white matter streamlines using open-source DWI data from 1065 healthy participants (Yeh et al., 2018; http://brain.labsolver.org/diffusion-mri-templates/hcp-842-hcp-1021), as performed before (Mitsuhashi et al., 2021, 2022; Sonoda et al., 2021; Ono et al., 2022). We placed seeds at cortical ROIs revealing significant high-gamma co-augmentation lasting ≥100 ms. The DSI Studio script (http://dsi-studio.labsolver.org/) visualized tractography streamlines directly connecting the ROIs within the Montreal Neurological Institute (MNI) standard space. The following values were parameters used for fiber tracking: a quantitative anisotropy threshold of 0.05, a maximum turning angle of 70°, a step size of 0 mm, and a streamline length of 20 to 250 mm. We excluded streamlines involving the brainstem, basal ganglia, thalamus, or cerebrospinal fluid space from the present dynamic tractography analysis. The resulting dynamic tractography video atlases visualized functional connectivity modulations, at each 100-ms epoch in 10-ms sliding windows.

**Fig. 5.**
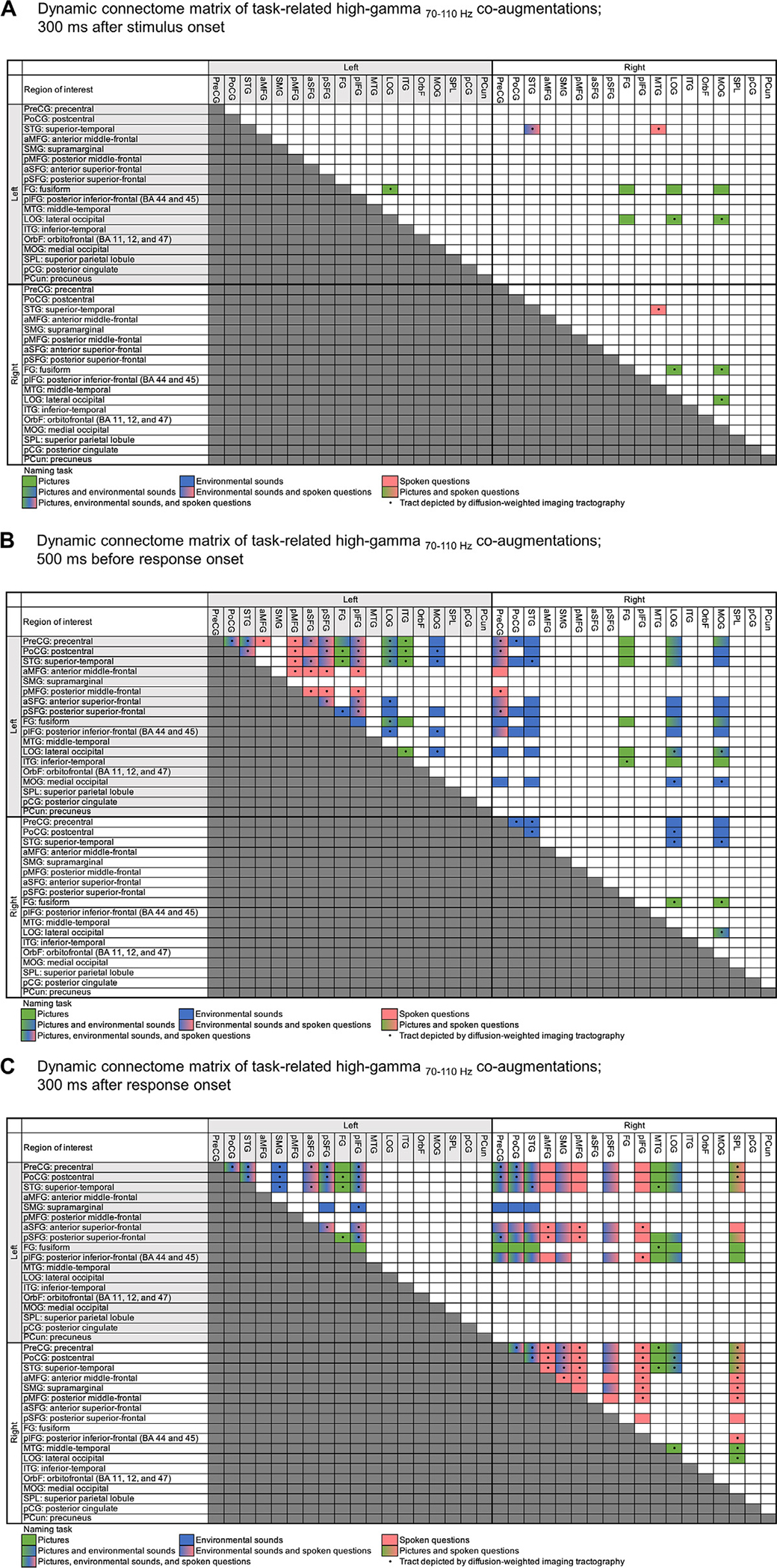
Snapshots of dynamic connectome matrix. The color of each box indicates the given naming task(s) that elicited significant direct functional connectivity enhancement between the two corresponding regions of interest (ROIs) at (A) 300 ms after stimulus onset. (B) 500 ms before response onset. (C) 300 ms after response onset. Functional connectivity was considered enhanced when two ROIs linked via direct DWI streamlines showed significant (based on the studentized bootstrap statistics), simultaneous, and sustained (≥100 ms) high-gamma co-augmentation. Light green: picture naming. Blue: non-speech environmental sound naming. Magenta: auditory descriptive naming. Video S5 shows the dynamic connectome matrix as a function of time.

**Fig. 6.**
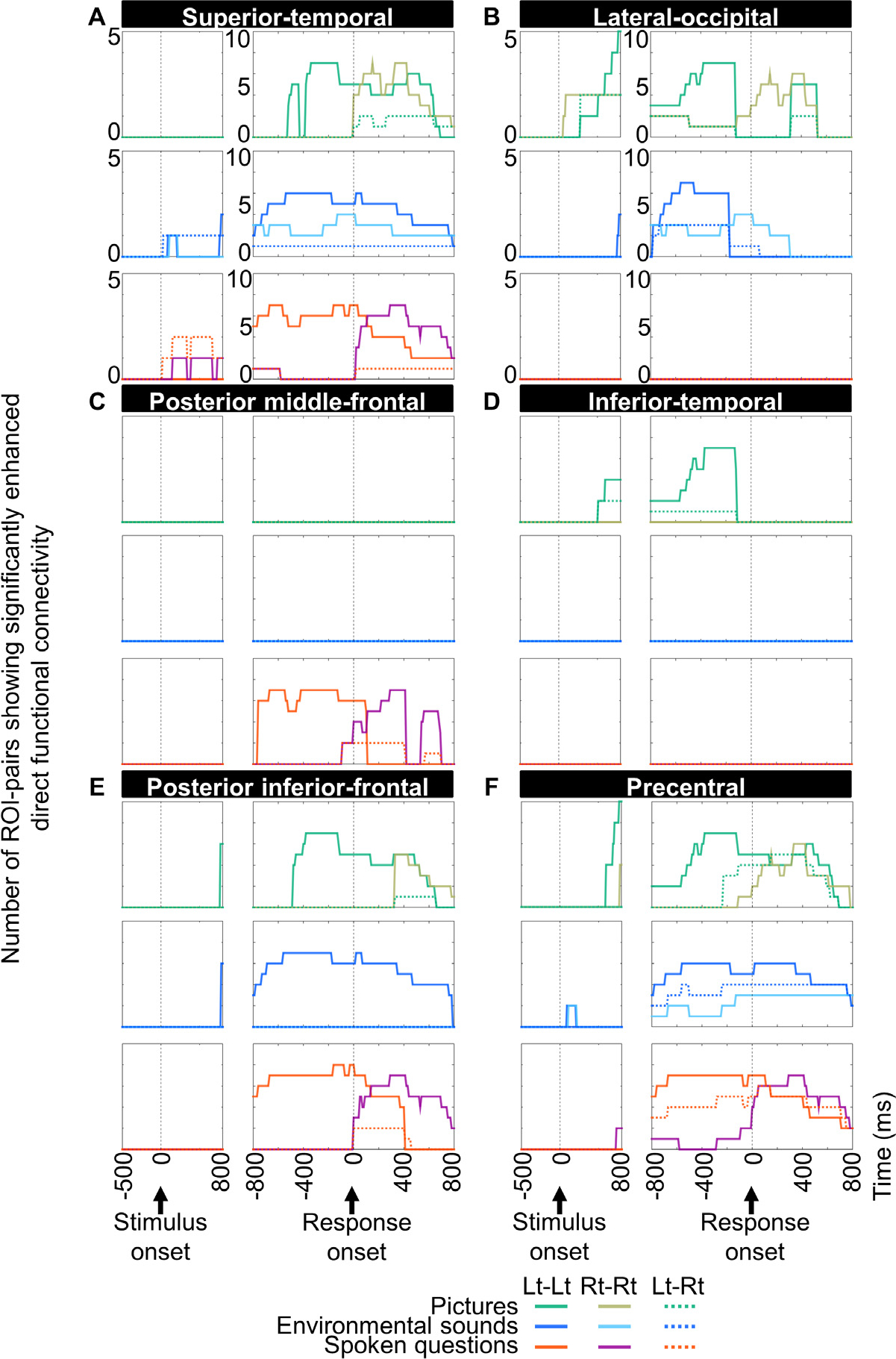
Dynamic change of intra- and inter-hemispheric direct functional connectivity. Each plot indicates the number of regions of interest (ROI)-pairs showing significantly enhanced direct functional connectivity. Solid line: intra-hemispheric connectivity enhancement. Broken line: inter-hemispheric connectivity enhancement. Green and tea green: picture naming. Blue and light blue: nonspeech environmental sound naming. Magenta and purple: auditory descriptive naming. (A) Superior temporal gyrus. (B) Lateral occipital gyrus. (C) Posterior middle frontal gyrus. (D) Inferior temporal gyrus. (E) Posterior inferior frontal gyrus. (F) Precentral gyrus. Video S6 shows the connectivity data from all ROIs.

We generated a dynamic connectome matrix visualizing the spatiotemporal characteristics of cortical ROI-pairs showing significant functional connectivity enhancement, during each time period for each task (Fig. 5). We also plotted the number of ROI-pairs showing significantly enhanced direct functional connectivity via intra- and inter-hemispheric white matter pathways from each ROI as a function of time (Fig. 6). Videos S5 and S6 present results of the whole-brain level analyses for all time windows. These figures and videos were designed to help readers visually assess the spatio-temporal dynamics of task-specific connectivity enhancement patterns.

**Fig. 7.**
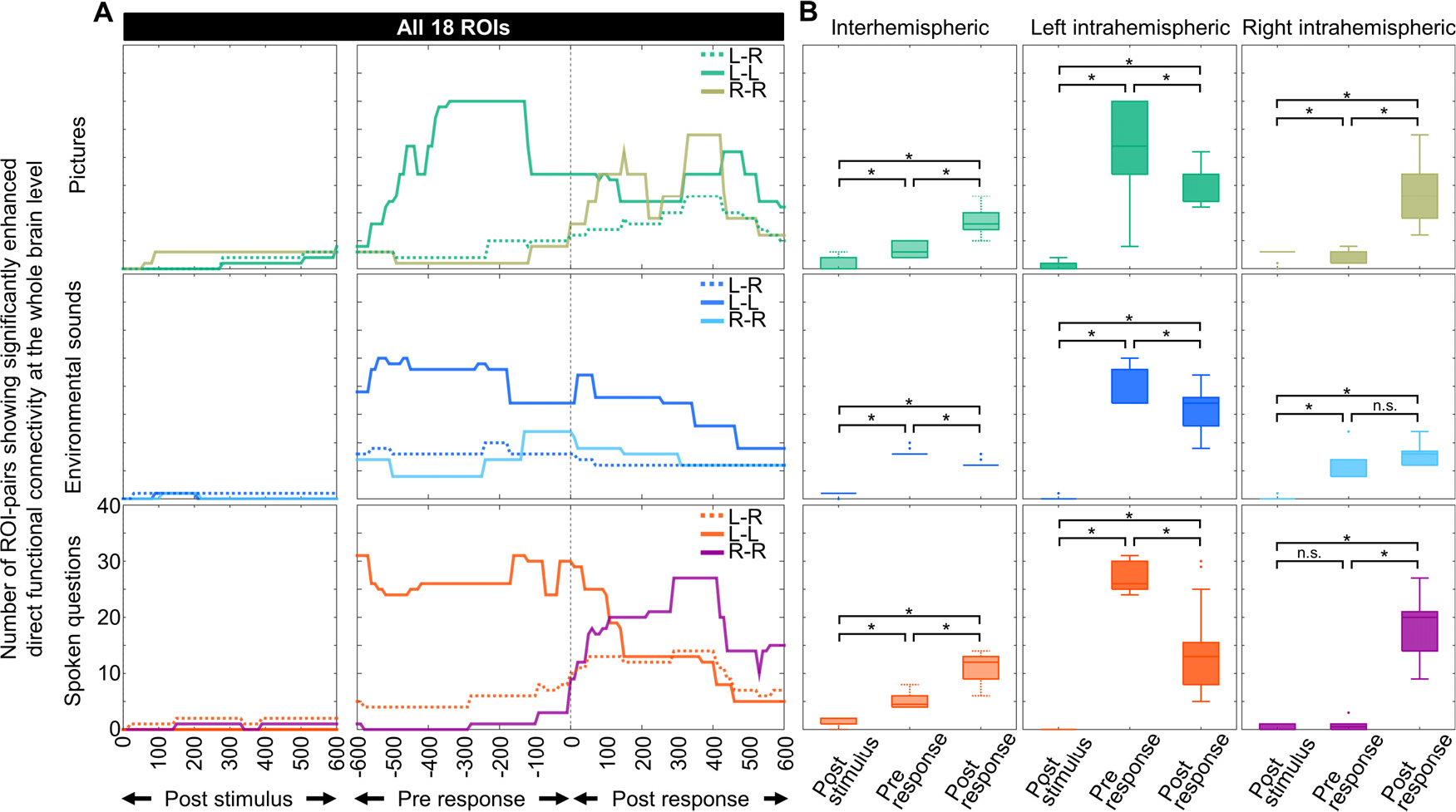
The spatial extent of intra- and inter-hemispheric direct white matter connectivity enhancements. (A) Number of regions of interest (ROI)-pairs showing significantly enhanced direct functional connectivity during each of the three 600-ms periods (unit: number of ROI-pairs/ms; left ‘post stimulus’: after stimulus onset; middle ‘pre response’: before response onset; right ‘post response’: after response onset). Solid line: intra-hemispheric connectivity enhancement. Broken line: inter-hemispheric connectivity enhancement. (B) Median number of ROI-pairs showing significantly enhanced direct functional connectivity during of the three 600-ms periods (unit: number of ROI-pairs/ms; left: inter-hemispheric; middle: left intra-hemispheric; right: right inter-hemispheric). Upper row: picture naming (green and tea green); middle row: nonspeech environmental sound naming (blue and light blue); bottom row: auditory descriptive naming (magenta and purple). Each box shows the 25 to 75th percentiles, with a horizontal line at the median. Whiskers indicate the highest and lowest values no further than the 1.5 × interquartile range. Dots show outliers. ^∗^: significant difference based on a two-sided uncorrected p-value of <0.0056 on the Wilcoxon signed-rank test.

To further characterize the dynamics of intra- and inter-hemispheric connectivity enhancements in each naming task, we determined whether the spatial extent of intra- and inter-hemispheric connectivity differed across the following three 600-ms periods: [1] immediately after stimulus onset, [2] immediately before response onset, and [3] immediately after response onset. We treated ‘the median number of ROI-pairs showing enhanced intra-hemispheric connectivity during each 600-ms period’ as the summary measure of the spatial extent of intra-hemispheric connectivity enhancements (unit: number of ROI-pairs/ms). The Friedman test followed by a pairwise comparison using the Wilcoxon signed-rank test determined whether the spatial extent of intra-hemispheric connectivity enhancements differed between the three periods (Fig. 7). We likewise determined whether the spatial extent of inter-hemispheric connectivity enhancements differed between the three periods.

### 2.10. Judging the effects of potential biases between patients

We employed an ancillary mixed model analysis to determine whether ‘patient age’ or ‘response time’ was independently associated with naming-related high-gamma modulations. The dependent variable was high-gamma amplitude averaged within each 300-ms period of interest, at a given ROI during a given naming task. The fixed effect predictor variables included [a] sampled hemisphere (1 if the right hemisphere), [b] age (years), and [c] median response time (seconds; Table 1). We employed an FDR correction because of the repeated testing across ten periods of interest (i.e., first and second 300-ms immediately after stimulus onset, immediately before/after stimulus offset, and immediately before/after response onset) and 18 ROIs.

## 3. Results

### 3.1. Behavioral results

#### 3.1.1. Correct response rate

The median correct response rate across 13 patients was 0.95 (range: 0.83 to 1) in picture naming, 0.82 (range: 0.68 to 0.92) in nonspeech environmental sound naming, and 0.97 (range: 0.80 to 0.98) in auditory descriptive naming. The Friedman test demonstrated that the correct response rate differed among three naming tasks (*p*<0.001; Chi-square value=16.62). The post-hoc Wilcoxon signed-rank test suggested that nonspeech environmental sound naming was associated with a lower response rate, compared to picture naming (*p* = 0.001; Z-value=−3.18; *r* = 0.62 on the Wilcoxon signed-rank test) and auditory descriptive naming (*p* = 0.002; Z-value=−3.11; *r* = 0.61 on the Wilcoxon signed-rank test).

#### 3.1.2. Response time

The median response time across 13 patients was 1.34 s (range: 0.86 to 2.03) in picture naming, 2.63 s (range: 2.05 to 3.84) in environmental sound naming, and 3.27 s (range: 2.52 to 4.13) in auditory descriptive naming. It should be noted that a substantial proportion of patients were able to identify objects before the stimulus offset, during the picture naming task. The Friedman test demonstrated that the response time differed among the three naming tasks (*p<*0.001; Chisquare value = 22.62). The post-hoc Wilcoxon signed-rank test suggested that picture naming was associated with a shorter response time than nonspeech environmental sound naming (*p* = 0.001; Z-value=−3.18; *r* = 0.62 on the Wilcoxon signed-rank test), whereas nonspeech environmental sound naming was associated with a shorter response time than auditory descriptive naming (*p* = 0.003; Z-value=−2.97; *r* = 0.58 on the Wilcoxon signed-rank test).

### 3.2. Type I error estimate in evaluating high–gamma co–augmentation

The iEEG time-frequency analysis revealed significant high-gamma augmentation in 21.9% of the 4700-ms analysis time window, on average, across 36 ROIs (picture: 22.5%; environmental sound: 24.3%; auditory descriptive naming: 19.2%). The Type I error of finding significant high-gamma co-augmentation lasting ≥100 ms was estimated to be ≈ 0.000000019.

### 3.3. High–gamma co–augmentation regions accompanied and unaccompanied by direct white matter streamlines

The dynamic connectome matrix indicated that only some ROI-pairs showing high-gamma co-augmentation on iEEG recording demonstrated direct white matter streamlines on DWI tractography (Fig. 5; Video S5). Specifically, 66 out of the 78 left-hemispheric (84.6%) and 47 out of the 68 right-hemispheric ROI-pairs (69.1%) showing significant high-gamma co-augmentation were accompanied by direct intra-hemispheric white matter streamlines on DWI tractography. In contrast, only 24 out of the 130 left-to-right ROI-pairs (18.5%) were accompanied by direct inter-hemispheric white matter streamlines.

### 3.4. Dynamics of intra– and inter–hemispheric direct connectivity enhancements

The spatial extent of left intra-hemispheric and inter-hemispheric connectivity enhancements was smallest during the 600-ms period after stimulus onset. Left intra-hemispheric connectivity enhancements were most extensive during the 600-ms period before response. Inter-hemispheric connectivity enhancements were most extensive during the 600-ms period before response in nonspeech environmental sound naming and during the 600-ms period after response in picture and auditory descriptive naming tasks. Fig. 7 summarizes the statistical results describing spatial extent differences across time.

In the picture naming task, for example, the spatial extent of left intra-hemispheric connectivity enhancements differed across time periods (median: 1 ROI-pair/ms after stimulus onset; 22 ROI-pairs/ms before response onset, and 17 ROI-pairs/ms after response onset (*p* = 0.000; Chi-square value = 100.20 on the Friedman test). The left intra-hemispheric connectivity enhancements were more extensive before response onset than after response onset (*p*<0.001; Z-value = 4.248; *r* = 0.39 on the Wilcoxon signed-rank test) and more extensive after response onset than after stimulus onset (*p*<0.001; Z-value = 6.771; *r* = 0.62 on the Wilcoxon signed-rank test). Likewise, the spatial extent of inter-hemispheric connectivity enhancements differed across time periods during picture naming (median: 2 ROI-pairs/ms after stimulus onset; 3 ROI-pairs/ms before response onset, and 8 ROI-pairs/ms after response onset (*p* = 0.000; Chi-square value = 115.42 on the Friedman test). The inter-hemispheric connectivity enhancements were more extensive after response onset than before response onset (*p*<0.001; Z-value = 6.700; *r* = 0.61 on the Wilcoxon signed-rank test) and more extensive before response onset than after stimulus onset (*p*<0.001; Z-value = 6.414; *r* = 0.59 on the Wilcoxon signed-rank test).

### 3.5. Network dynamics supporting picture naming

#### 3.5.1. Assessment time–locked to stimulus onset

During the 500-ms period immediately after stimulus onset, intra- and inter-hemispheric connectivity enhancements were confined across the lateral/medial occipital and fusiform regions (Video S1). The bilateral lateral, as well as right medial, occipital and fusiform regions showed significantly enhanced intra-hemispheric connectivity via the inferior longitudinal fasciculus and increased inter-hemispheric connectivity via posterior callosal fibers by +280 ms post-stimulus onset. Subsequently, the left arcuate fasciculus between the inferior temporal gyrus (ITG) and precentral gyrus and the left superior longitudinal fasciculus between the lateral occipital and precentral gyri were significantly enhanced by +600 ms post-stimulus onset.

#### 3.5.2. Assessment time–locked to response onset

The left intra-hemispheric connectivity enhancements propagated in a posterior-to-anterior direction between −800 and −120 ms pre-response onset (Video S1). The left precentral gyrus showed significantly enhanced connectivity with the lateral occipital gyrus via the superior longitudinal fasciculus and with the ITG via the arcuate fasciculus, at −800 ms pre-response onset. The left posterior inferior frontal gyrus (pIFG) showed enhanced connectivity with: [1] the lateral occipital gyrus via the inferior fronto-occipital fasciculus at −480 ms pre-response onset, [2] the ITG via the arcuate fasciculus at −480 ms, [3] the precentral gyrus via the frontal U-fibers at −480 ms, and [5] the STG via the arcuate fasciculus at −380 ms. Among the findings mentioned above, enhancements of the left arcuate fasciculus between the ITG and precentral/pIFG were specific to the picture naming task (Video S5).

Subsequently, the precentral, postcentral, and superior temporal gyri showed inter-hemispheric connectivity enhancements. The precentral and postcentral gyri showed enhanced inter-hemispheric connectivity via the mid-callosal fibers by −110 ms pre-response onset. The STG showed enhanced inter-hemispheric connectivity via the posterior callosal fibers at response onset. In turn, the inter-hemispheric connectivity enhancements between left lateral and right medial/lateral occipital regions subsided by −130 ms (Video S1).

### 3.6. Network dynamics supporting nonspeech environmental sound naming

#### 3.6.1. Assessment time-locked to stimulus onset

During the 780-ms period immediately after stimulus onset, intra- and inter-hemispheric connectivity enhancements commonly involved the STG (Video S2). Specifically, the bilateral STG began to show inter-hemispheric connectivity enhancement via the posterior callosal fibers at +30 ms; this inter-hemispheric connectivity enhancement was continuous during listening and took place again around response onset. The arcuate fasciculus between STG and precentral gyri in each hemisphere was enhanced by +110 ms post-stimulus onset.

Subsequently, the left inferior longitudinal fasciculus between the STG and lateral occipital gyrus and the left inferior fronto-occipital fasciculus between the lateral occipital and pIFG were enhanced by +780 ms. The left arcuate fasciculus between the STG and pIFG was enhanced at +780 ms.

#### 3.6.2. Assessment time–locked to stimulus offset

The left intra-hemispheric connectivity enhancements propagated in a posterior-to-anterior direction around stimulus offset (Video S2). The left inferior longitudinal fasciculus between the STG and lateral/medial occipital gyri and the left inferior fronto-occipital fasciculus between the pIFG and lateral/medial occipital gyri were enhanced by −280 ms pre-stimulus offset. The bilateral occipital regions showed enhanced inter-hemispheric connectivity via the posterior callosal fibers by −280 ms. The left frontal aslant fasciculus between the anterior superior frontal gyrus (aSFG) and pIFG and the superior frontal longitudinal fasciculus between the aSFG and precentral gyrus were enhanced by −190 ms. The left arcuate fasciculus between the STG and precentral/pIFG was continuously enhanced before and after stimulus offset.

#### 3.6.3. Assessment time–locked to response onset

A posterior-to-anterior propagation of the left intra-hemispheric connectivity enhancements was noted between −800 ms pre-response and +60 ms post-response onset (Video S2). The left inferior longitudinal and inferior fronto-occipital fasciculi involving the left lateral/medial occipital gyri were enhanced by −690 ms pre-response and subsided by +60 ms post-response onset. The left frontal aslant and the superior frontal longitudinal fasciculi across the aSFG and pIFG/precentral gyrus were enhanced by −690 ms pre-response onset. Among the findings mentioned above, enhancement of the left inferior longitudinal and inferior fronto-occipital fasciculi between medial occipital and STG/pIFG was specific to the nonspeech environmental sound naming task (Video S5). The left arcuate fasciculus and frontal U-fibers across the STG, pIFG, and precentral gyrus were continuously enhanced before and after response onset.

The precentral, postcentral, and STG showed enhanced inter-hemispheric connectivity around response onset, as observed during the picture naming task.

### 3.7. Network dynamics supporting auditory descriptive naming

#### 3.7.1. Assessment time–locked to stimulus onset

During the 800-ms period immediately after stimulus onset, intra- and inter-hemispheric connectivity enhancements commonly involved the STG (Video S3). The bilateral STG began to show significant inter-hemispheric connectivity enhancement via the posterior callosal fibers at +20 ms post-stimulus onset; this inter-hemispheric connectivity enhancement was continuous during stimulus listening and took place again after response onset. The white matter fibers between the right STG and middle temporal gyrus (MTG) were significantly enhanced at +150 ms, whereas those between the right STG and precentral gyri were enhanced by +730 ms.

#### 3.7.2. Assessment time–locked to stimulus offset

The left intra-hemispheric connectivity enhancements propagated in a posterior-to-anterior direction around stimulus offset (Video S3). The left inferior longitudinal fasciculus between the STG and lateral occipital gyrus was enhanced between −410 ms pre-stimulus offset and +140 ms post-stimulus offset. The left STG showed enhanced connectivity with the MTG via the U-fibers in period between −130 ms and +210 ms. The left MTG showed enhanced connectivity with the pIFG, aSFG, and precentral gyrus via the arcuate and uncinate fasciculi between +90 and +250 ms. The pIFG, aSFG, and precentral gyrus showed enhanced connectivity with the anterior/posterior middle frontal gyrus (aMFG/pMFG) via the frontal U-fibers and superior frontal longitudinal fasciculus between +270 and +800 ms.

#### 3.7.3. Assessment time–locked to response onset

The left arcuate fasciculus and frontal U-fibers across the STG, aSFG, pIFG, aMFG, pMFG, and precentral gyrus were continuously enhanced before response onset. The connectivity enhancements involving the left aSFG, pIFG, aMFG, and pMFG subsided by +360 ms post-response onset. Among the findings mentioned above, enhancements of the left arcuate fasciculus and frontal U-fibers involving the aMFG and pMFG were specific to the auditory descriptive naming task (Video S5).

The precentral, postcentral, and superior temporal gyri showed enhanced inter-hemispheric connectivity around response onset, as observed during the other naming tasks.

### 3.8. Judging the effects of potential biases between patients

The ancillary mixed model analysis indicated a positive effect of patient age on high-gamma amplitude at the superior-temporal region during the 300-ms period immediately before response onset in the environmental sound naming task (mixed model estimate =+1.15% [95% confidence interval=+0.57 to +1.74%]; t-value=+3.9). This finding infers that each one-year increase in age was associated with a 1.15% increase in high-gamma amplitude. No other ROIs showed a significant age effect.

The ancillary mixed model analysis also indicated a positive effect of response time on high-gamma amplitude at the orbitofrontal region during a 300-ms period immediately after stimulus onset in the auditory description naming task (mixed model estimate= +4.31% [95% confidence interval=+2.20 to +6.42%]; t-value=+4.1). This finding infers that each 1-second increase in response time was associated with a 4.31% increase in high-gamma amplitude. No other ROIs showed a significant response time effect.

## 4. Discussion

### 4.1. Novelty

To our knowledge, this is the first study to provide video atlases animating the naming-related network dynamics through direct intra- and inter-hemispheric white matter tracts (Videos S1–S3). The study significantly improved the currently available models of object recognition and speech production by adding the dynamics of coordinated functional iEEG activity via direct white matter pathways. We estimated the enhancement of direct functional connectivity using the concept that cortical regions with significant, simultaneous, and sustained high-frequency neural responses beyond a chance level are likely involved in a coordinated interaction (Singer, 1993, 2018; Buonomano and Merzenich, 1998). This concept has been widely employed in previous fMRI, magnetoencephalography (MEG), scalp EEG, and iEEG studies (Rissman et al., 2004; Sun et al., 2004; Fox and Greicius, 2010; Palva and Palva, 2012; Crossley et al., 2013; Wehrl et al., 2013; Cohen, 2014; Glasser et al., 2016; Beldzik et al., 2019; Grappe et al., 2019; Jamadar et al., 2019). Our previous iEEG study provided the initial empirical data in which distant cortical regions showing simultaneous task-related high-gamma augmentation were accompanied by in-creased effective connectivity rated by single-pulse electrical stimulation (Sonoda et al., 2021).

The innovative strength of the current iEEG study is including the presence of biologically-plausible direct white matter streamlines on DWI tractography to define direct functional connectivity enhancement. For example, we believe it implausible to assume that the right occipital and left frontal regions can directly interact via monosynaptic white matter pathways, even if these regions showed significant, simultaneous, and sustained neural activation during naming tasks. Our empirical data indicated that only 18.5% of inter-hemispheric ROI-pairs showing significant, simultaneous, and sustained high-gamma augmentation were accompanied by direct white matter streamlines (Video S5). In other words, functional connectivity in the present study was a measure distinct from high-gamma co-modulations.

Another innovation of this study is using animation to describe the neurobiological models of object recognition and speech production, as conventional models are typically summarized in still images or schematic illustrations. To allow readers to readily appreciate the rapidly-alternating large-scale neural dynamics at cortical and white matter levels, we designed each movie atlas to animate white matter connectivity modulations in 10-ms sliding windows together with local, cortical high-gamma modulations (Videos S1–S3). The resulting atlases delineated the network dynamics common across naming tasks, characterized by (i) intra- and inter-hemispheric connectivity enhancements directly from the perceptual areas right after stimulus onset, (ii) left-hemispheric intra-hemispheric connectivity enhancements propagating in a posterior-to-anterior direction during response preparation, and (iii) intra- and inter-hemispheric connectivity enhancements across sensorimotor and auditory areas during overt responses. We believe our video atlases will be valuable for helping lay audiences, clinicians, and neuroscientists understand the neurobiological mechanisms of object recognition and speech production.

### 4.2. Neurobiological model of object recognition and speech production

#### 4.2.1. Visual perceptual processing

The early neural dynamics observed after picture stimulus onset were consistent with the currently available models of visual object recognition (Logothetis and Sheinberg, 1996; Grill-Spector et al., 2001; Palmeri and Gauthier, 2004; DiCarlo et al., 2012). The present study showed that high-gamma activity was augmented in the right medial/lateral occipital regions by +60 ms, the bilateral fusiform regions by +140 ms, and the left lateral occipital region by +280 ms post-stimulus onset. The spatial extent of intra- and inter-hemispheric connectivity enhancements were confined across these lower- and higher-order visual cortices during the first 500 ms after stimulus onset, and the connectivity enhancements during this period were much less extensive, compared to during response preparation. These observations are consistent with the notion that conscious visual perception is computed within the lower- and higher-order visual cortices through local, short-range connections and via the long-range inter-hemispheric white matter pathways.

A novel observation of the current study includes the timing of inter-hemispheric connectivity enhancements across visual cortices. Previous lesion-deficit studies demonstrated the causal significance of inter-hemispheric callosal pathways in object recognition (Lausberg et al., 2003; Bloom and Hynd, 2005; Gazzaniga, 2005) but were not designed to disclose the exact timing of inter-hemispheric coordination, during a given task. The current study found that picture stimuli significantly enhanced inter-hemispheric connectivity via the posterior callosal fibers within +280 ms after stimulus onset (Video S1). Based on onset latency, the observed inter-hemispheric connectivity enhancements can be at least partially attributed to inter-hemispheric coordination required for integrating mental representations of visual features continuously processed bilaterally. It is plausible to expect that such inter-hemispheric coordination allows perceptual and subsequent semantic recognition of the complete picture of a given stimulus.

In turn, the inter-hemispheric connectivity enhancements across the left and right lower- and higher-order visual areas subsided −130 ms pre-response onset (Fig. 6). Such alternating inter-hemispheric connectivity enhancement and subsidence patterns are consistent with the notion that perceptual-motor resources are not unlimited (Desimone and Duncan, 1995; Meyer and Kieras, 1997), and the observed connectivity patterns may account for the temporary reduction of visual attention during overt speech (e.g., an unwanted observation during car driving; Horrey and Wickens, 2006).

#### 4.2.2. Auditory perceptual processing

The early neural dynamics observed after auditory stimulus onset likely represent processes including conscious auditory perception and sound-motor transformation. In both auditory-related naming tasks, high-gamma augmentation involved the bilateral STG by +30 ms post-stimulus onset, continuously lasted until stimulus offset and showed up around response onset (Videos S2 and S3). We hypothesize that such STG activation includes coordinated processing within the STG through local, short-range connections (Trumpis et al., 2021; Norman-Haignere et al., 2022). Inter-hemispheric connectivity enhancements between the bilateral STGs via the posterior callosal fibers were continuously observed between 30 ms post-stimulus onset and 230 ms post-stimulus offset. It is plausible to expect that such inter-hemispheric coordination allows precise temporal integration for a coherent perception (Hipp et al., 2011; Steinmann et al., 2014). In the nonspeech environmental sound naming task, the arcuate fasciculus between the STG and precentral gyri in either hemisphere was enhanced between +90 and +210 ms post-stimulus onset. These regions are suggested to be a part of the phonological loop structures, and early arcuate fasciculus enhancements during stimulus listening may support sound-motor transformation, in part, via subvocal rehearsal (Paulesu et al., 1993; Baddeley et al., 2003; Pulvermüller et al., 2006; Rauschecker and Scott, 2009; Price, 2010; Cogan et al., 2014; Nishida et al., 2017). The intra- and inter-hemispheric connectivity enhancements were confined to pathways involving the STG during the first 780 ms after stimulus onset; connectivity enhancements during this period were much less extensive than during response preparation. Such a long absence of measurable connectivity enhancements outside the perceptual areas can be attributed partly to the stimulus design. In the auditory descriptive naming task, a given sentence was initiated not with a concrete word but with a *wh*-interrogative (e.g. *who, what, when, where*). The response time was longer in auditory-related than picture naming, which is consistent with the notion that auditory-related naming tasks require more extended perceptual processing than picture naming.

#### 4.2.3. Semantic and lexical retrieval in picture naming

We suggest that picture naming-related enhancements of the left intra-hemispheric direct connectivity pathways between −450 and −150 ms pre-response onset reflect the network dynamics supporting semantic and lexical retrieval. This notion is consistent with the previously proposed neurobiological models of speech production, as described below. The dorsal connectivity pathways enhanced during this 300-ms response preparation period include the left arcuate fasciculus between the ITG and precentral/pIFG and the left superior longitudinal fasciculus between the lateral occipital and precentral gyri. Many lesion-deficit and electrical stimulation studies have suggested that these left-hemispheric dorsal pathways play an essential role in lexical retrieval (Hickok and Poeppel, 2007; Glasser and Rilling, 2008; Ellmore et al., 2009; Rauschecker and Scott, 2009; Catani et al., 2012; Dick et al., 2014; Gierhan, 2013; Han et al., 2013; Chang et al., 2015; Skeide and Friederici, 2016; Gajardo-Vidal et al., 2021).

The current study showed that enhancements of the arcuate fasciculus between the ITG and precentral/pIFG during the response preparation period were specific to picture naming (Fig. 5); thus, a plausible hypothesis is that this ITG-based portion of the arcuate fasciculus may be part of the visual modality-preferential lexical retrieval pathways. Our previous iEEG study reported that electrical stimulation of the basal temporal lobe, including the inferior temporal region, preferentially elicited errors in picture naming; in contrast, stimulation of the STG and MTG elicited errors in auditory descriptive naming (Sonoda et al., 2022).

The ventral connectivity pathways enhanced during response preparation included the left inferior fronto-occipital fasciculus between the lateral occipital and pIFG. Investigators reported that electrical stimulation of the left inferior fronto-occipital fasciculus induced semantic paraphasia (Duffau et al., 2005; Bello et al., 2008); thus, semantic processing is likely among the functional roles of the left inferior fronto-occipital fasciculus (Martino et al., 2010).

#### 4.2.4. Semantic and lexical retrieval in nonspeech environmental sound naming

We suggest that the left intra-hemispheric connectivity enhancements occurring around stimulus offset and between −700 to −200 ms pre-response onset reflect the network dynamics supporting semantic and lexical retrieval in environmental sound naming. Lesion deficit and electrical stimulation mapping studies suggest that the left arcuate fasciculus between the STG and precentral/pIFG is among the critical, enhanced structure observed in the present study (Gajardo-Vidal et al., 2021; Nakai et al., 2017; Sonoda et al., 2022). Notably, posterior-dominant connectivity enhancements in the left inferior longitudinal and inferior fronto-occipital fasciculi between the medial/lateral occipital and STG/pIFG were prominent in nonspeech environmental sound naming but not in auditory descriptive naming. We hypothesize that these connectivity enhancements support mental scanning of visual imagery for facilitating semantic retrieval. A meta-analysis of 297 studies reported a double dissociation characterized by spoken sound-preferential responses localized in the bilateral superior temporal gyrus (STG) and environmental sound-preferential responses in the high-order visual cortices for mental scanning of visual imageries (Schirmer et al., 2012). This hypothesis is supported by the general observation that wild non-human primates use their ears to rapidly recognize and respond to predators and prey, as well as challenging and advantageous situations (Zuberbühler et al., 2022).

#### 4.2.5. Semantic and lexical retrieval in auditory descriptive naming

The left intra-hemispheric connectivity enhancements involving MTG/STG around stimulus offset likely support semantic and lexical retrieval in auditory descriptive naming. The left arcuate fasciculus enhancements between the STG/MTG and precentral/pIFG may be among the most critical of all observed connectivity enhancements (Hamberger et al., 2001; Tate et al., 2014; Nakai et al., 2017; Gajardo-Vidal et al., 2021; Sonoda et al., 2022). Notably, enhancements of the left arcuate fasciculus and frontal U-fibers involving the MTG and pMFG were specific to auditory descriptive naming. It is plausible to hypothesize that these connectivity enhancements play a critical role in syntactic processing, which is a unique human function. Our previous study of 100 epilepsy patients reported that stimulation of the left MTG and pMFG frequently impaired responses during auditory descriptive naming due to failure of either understanding the questions or retrieving the relevant word (Nakai et al., 2017).

#### 4.2.6. Phonological retrieval

It is plausible to hypothesize that phonological retrieval is partly supported by enhancements of left-hemispheric u-fibers and arcuate fasciculus across the STG, pIFG, and precentral gyri because these networks were found to be enhanced by −300 ms pre-response, in all three naming tasks. The white matter pathways connecting these three regions are suggested to be a part of the phonological loop and involved in the maintenance and retrieval of mental representations of phonological information (Herman et al., 2013; Hagoort and Indefrey, 2014; Flinker et al., 2015; Papagno et al., 2017; Nishida et al., 2017).

Our current results do not suggest that phonological retrieval is directly supported by the left arcuate fasciculus between the ITG and precentral/pIFG or the left superior longitudinal fasciculus between the lateral occipital and precentral gyri. These specific portions of the left-hemispheric fasciculi were not enhanced before overt responses in auditory descriptive naming, though this task likewise requires phonological retrieval.

#### 4.2.7. Overt articulation and monitoring of own responses

Inter-hemispheric connectivity between sensorimotor areas via the corpus callosum began enhancement by −100 ms pre-response onset and it was maximized 250 ms after the response; this enhancement may support the synchronous and symmetric movements of oral and vocal structures (Hoptman and Davidson, 1994; Hervé et al., 2013). In turn, enhanced intra-hemispheric connectivity across precentral, postcentral, and superior temporal gyri, as well as inter-hemispheric connectivity between the bilateral STGs, around response onset may support monitoring of one’s own overt responses (Zheng et al., 2010; Greenlee et al., 2013).

### 4.3. Methodological limitations

We sought to reduce the false positive detection of task-related connectivity enhancements by incorporating tractography criteria. Thus, it is plausible to hypothesize that our analytic approach increased the false negative detection of true connectivity enhancement. Delineation and validation of short-range U-fiber streamlines are especially challenging due to known crossing fibers and the lack of any gold standard (Wang et al., 2012; Movahedian Attar et al., 2020). Thus, in the present study, we assumed that sites within the same gyrus showing high-gamma co-augmentation (e.g., STG during sound listening) may have been connected via local, short-range white matter pathways (Trumpis et al., 2021; Norman-Haignere et al., 2022).

We should interpret the following DWI observations cautiously due to the inherent limitations of tractography-based visualization of white matter pathways (Daducci et al., 2016). The present study revealed that only 24 of the 130 inter-hemispheric ROI-pairs showing significant, sustained high-gamma co-augmentation (18.5%) were accompanied by direct inter-hemispheric white matter streamlines. In contrast, 66 out of the 78 left-hemispheric (84.6%) and 47 out of the 68 right-hemispheric ROI-pairs (69.1%) showing such high-gamma co-augmentation were accompanied by direct intra-hemispheric streamlines. While a portion of this difference may reflect legitimate anatomy, one also has to consider that inherent limitations with tractography may contribute, as well. For example, inter-hemispheric streamlines are typically longer than intra-hemisphereic ones. A longer distance between ROIs increases susceptibility to failed detection of white matter pathways due to the crossing fiber problem. Thus, we cannot rule out the possibility that this issue partly accounts for our observed discrepancy between inter- and intra-hemispheric streamline detection.

We have previously validated the multimodal analysis combining iEEG measures from nonepileptic cortices and DWI data from healthy subjects (Sonoda et al., 2021). In that study, we computed the velocity of neural propagations elicited by single-pulse electrical stimulation (SPES) using individual and open-source DWI tractography data. We found that the propagation velocity computed with individual tractography data was highly correlated to that with open-source data (*Pearson’s r* = 0.8). This observation supports the notion that the anatomical trajectories of streamlines supporting given network dynamics are spatially similar between nonepileptic brain structures of individual patients and healthy participants in the human connectome project (Yeh et al., 2018). Many investigators have treated SPES-related neural propagations as a gold standard to define direct connectivity in vivo (Matsumoto et al., 2004).

We assessed the temporal dynamics of high-gamma modulations using the widely applied Desikan atlas-based ROIs (Desikan et al., 2006; Nakai et al., 2017). We were unable to reduce the size of ROIs because of the resultant technical difficulties for tractography and limited number of electrode sites for a given ROI. Sampling limitations are inevitable in any iEEG study, since all of our electrodes were placed strictly according to clinical necessity; in other words, we did not extend the iEEG spatial sampling for research purposes, and hence, some ROIs may have not included an ideal number of samples (Mercier et al., 2022). Lack of electrode sampling from the homotopic areas may have decreased the chances of finding significant high-gamma co-augmentation in a given inter-hemispheric ROI-pair. In the present study, the medial occipital gyrus, precuneus, superior parietal lobule, and anterior superior-frontal gyrus suffered from a lack of iEEG electrode sampling in some surface areas (Fig. 1). Among these ROIs, the medial occipital gyrus showed significant high-gamma co-augmentation during picture and environmental sound naming tasks (Figure 4; Video S4); still, the duration of high-gamma co-augmentation may have been potentially underestimated due to insufficient iEEG electrode coverage. We expect that the amount of sEEG data from deep cortices and thalamic nuclei will increase in the next decade (Kerezoudis et al., 2022). In the future, improved tractography sensitivity and increased sample size through collaboration may allow connectivity analysis across much smaller cortical/subcortical regions with satisfactory statistical power.

The present study was not designed to estimate the direction of neural propagations during a given task. Employment of Granger-type causality analysis may be warranted to visualize the directionality of enhanced connections (Flinker et al., 2015; Giahi Saravani et al., 2019).

The present study was conducted in a tertiary pediatric epilepsy center; thus, all patients were children. The significance of the age-dependent increase in high-gamma amplitude immediately before response onset during the environmental sound naming task is uncertain. The effect of patient age on network dynamics can only be properly assessed with a much larger sample size. Failure to find significant effects of patient age or response time on naming-related high-gamma amplitude at most ROIs can be attributed to the small sample size available for the ancillary mixed model analysis (Table S1). DWI studies of healthy individuals have suggested that white matter development continues in the prefrontal regions even during late adolescence (Asato et al., 2010; Lebel et al., 2018; Baum et al., 2022). In turn, our previous iEEG study of 32 patients ranging from 0.7 to 28.6 years old individuals revealed that high-gamma responses to nonspeech sounds lasted for a shorter time as a function of age and that older children and adults can more easily discard irrelevant nonspeech sounds (Sakakura et al., 2022). Collaborative studies in the future will give us an outstanding opportunity to clarify the developmental patterns of naming-related connectivity enhancements.

## Supplementary Material

7

Video S1

Video S2

Video S3

Video S4

Video S6

Video S5

1

3

2

6

4

5

## Figures and Tables

**Table 1:** Patient profile.

Patient^*a^	Age at surgery (years)	Sex	Sampled hemisphere	Seizure onset zone^*b^	Number of antiseizure drugs	Neuro-psychological score^*c^	Median response time in naming task (seconds)			Pathology ^*d^
							Environmental sounds	Spoken questions	Pictures	

1	13	M	Lt	Lt O	2 (LEV; LCM)	N/A	2.79	1.34	3.27	Dysplasia
2	16	F	Rt	N/A	2 (TPM; CLB)	130	2.46	0.86	3.02	N/A
3	14	F	Rt	Rt T	2 (LCM; LTG)	83	2.54	1.39	3.44	Tumor
4	15	F	Rt	Rt P	2 (ZNS; LTG)	97	3.15	1.25	3.08	Gliosis alone
5	19	F	Lt and Rt	Lt F	1 (OXC)	71	2.77	1.70	3.59	N/A
6	6	F	Lt	Lt P	1 (OXC)	N/A	2.63	1.49	3.11	Gliosis alone
7	19	M	Rt	Rt O	2 (LEV; OXC)	79	2.26	1.32	3.39	Dysplasia
8	14	F	Rt	Rt T	2 (OXC; LCM)	89	3.28	1.47	4.13	Dysplasia
9	16	M	Lt and Rt	N/A	1 (CLB)	102	2.05	1.16	2.52	N/A
10	10	M	Rt	Rt P	1 (LCM)	104	2.54	1.28	2.73	Dysplasia
11	19	M	Rt	Rt T	3 (OXC; LTG; CLB)	N/A	3.84	2.03	3.75	Dysplasia
12	15	M	Lt	Lt T	2 (VPA; LCM)	103	2.08	1.27	2.89	Gliosis alone
13	15	M	Lt and Rt	Lt T	1 (VPA)	91	3.12	1.62	3.33	Gliosis alone

F: Female. M: Male. Lt: Left. Rt: Right. F: Frontal. T: Temporal. O: Occipital. P: Parietal. CLB: Clobazam. LCM: Lacosamide.

LEV: Levetiracetam. LTG: Lamotrigine. OXC: Oxcarbazepine. TPM: Topiramate. VPA: Valproic acid. ZNS: Zonisamide.

*a : All patients were right-handed.

*b : N/A: Not available in patients #2 or 9 because no habitual seizure events were captured during the intracranial EEG recording.

*c : Patients #2–4, 7–10, 12, and 13: Peabody Picture Vocabulary Test. Patient #5: Verbal Comprehension Index.Patients #1, 6, and 11 attended regular school (7 th grade, 1st grade, and high school diploma, respectively).

*d : N/A: Not available in patients #2, 5, or 9, in whom resective surgery has not been performed.MRI failed to show a cortical lesion in these patients.A significant negative correlation was found between the Peabody Picture Vocabulary Test score and median response time in the auditory descriptive naming task (i.e., naming to spoken questions; False Discovery Rate [FDR]-corrected p-value = 0.038; Spearman’s rho = −0.78).

## Data Availability

The data can be made available upon reasonable request to the corresponding author. Investigators can find the codes used in the present study at https://github.com/kaz1126/flatten_map_and_tractography.
