# Supplementary material for: Intra– and inter–hemispheric network dynamics supporting object recognition and speech production
Source: Neuroimage. Author manuscript; Available in PMC 2023 Apr 18. (PMC10112006; doi:10.1016/j.neuroimage.2023.119954)
Supplement: 7 [file NIHMS1886331-supplement-7.docx]

**Supplementary document**

**in**

**Intra- and interhemispheric network dynamics**

**supporting object recognition and speech production**

Yu Kitazawa; Masaki Sonoda; Kazuki Sakakura; Takumi Mitsuhashi; Ethan Firestone; Riyo Ueda;

Toshimune Kambara; Hirotaka Iwaki; Aimee F. Luat; Neena I. Marupudi; Sandeep Sood; Eishi Asano

#: Y.K., M.S., and K.S. share the first authorship.

This document includes Table S1 and the legends for Videos S1-S6.

**Table S1. The exact number of artifact-free, nonepileptic electrode sites in given regions of interest.**

| **Region of interests** | **Left** | **Right** |
| --- | --- | --- |
| PreCG: precentral gyrus | 54 (5) | 87 (7) |
| PoCG: postcentral gyrus | 46 (6) | 49 (7) |
| STG: superior-temporal gyrus | 41 (6) | 45 (6) |
| aMFG: anterior middle-frontal gyrus | 26 (5) | 47 (8) |
| SMG: supramarginal gyrus | 24 (5) | 48 (7) |
| pMFG: posterior middle-frontal gyrus | 27 (4) | 43 (7) |
| aSFG: anterior superior-frontal gyrus | 13 (3) | 28 (6) |
| pSFG: posterior superior-frontal gyrus | 14 (3) | 12 (5) |
| FG: fusiform gyrus | 35 (6) | 29 (7) |
| pIFG: posterior inferior-frontal gyrus (BA 44 and 45) | 21 (4) | 38 (7) |
| MTG: middle-temporal gyrus | 22 (5) | 34 (6) |
| LOG: lateral occipital gyrus | 23 (3) | 31 (5) |
| ITG: inferior-temporal gyrus | 24 (6) | 26 (6) |
| OrbF: orbitofrontal region (BA 11, 12 and 47) | 22 (4) | 26 (7) |
| MOG: medial occipital gyrus | 16 (4) | 26 (5) |
| SPL: superior parietal lobule | 9 (2) | 13 (3) |
| pCG: posterior cingulate gyrus (posterior cingulate and isthmus cingulate) | 9 (3) | 12 (4) |
| PCun: precuneus | 9 (4) | 9 (4) |
| IPL: inferior parietal lobule | 4 (2) | 36 (6) |
| PCL: paracentral lobule | 4 (2) | 8 (4) |
| PHG: parahippocampal gyrus | 5 (3) | 5 (2) |
| Ent: entorhinal gyrus | 5 (4) | 3 (2) |
| aCG: anterior cingulate gyrus | 1 (1) | 2 (1) |
| TP: temporal pole | 1 (1) | 1 (1) |
| FP: frontal pole | 0 (0) | 1 (1) |
| **Total** | 455 (6) | 659 (10) |

Number in a parenthesis: number of contributing patients.

**Video S1. Cortical and functional connectivity modulations during picture naming.** Left: Dynamic cortical surface video presents the percent change of cortical high-gamma modulations, at a given epoch. Right: Dynamic tractography video presents the white matter pathways with significant intra-hemispheric (red) and inter-hemispheric (yellow) functional connectivity enhancement, at a given epoch.

**Video S2. Cortical and functional connectivity modulations during nonspeech environmental sound naming.** Left: Dynamic cortical surface video presents the percent change of cortical high-gamma modulations, at a given epoch. Right: Dynamic tractography video presents the white matter pathways with significant intra-hemispheric (red) and inter-hemispheric (yellow) functional connectivity enhancement, at a given epoch.

**Video S3. Cortical and functional connectivity modulations during auditory descriptive naming.** Left: Dynamic cortical surface video presents the percent change of cortical high-gamma modulations, at a given epoch. Right: Dynamic tractography video presents the white matter pathways with significant intra-hemispheric (red) and inter-hemispheric (yellow) functional connectivity enhancement, at a given epoch.

**Video S4. Cortical high-gamma modulations at regions of interest (ROIs).** A given plot presents the percent change in high-gamma amplitudes at each anatomical ROI compared to the baseline mean at 200 to 600 ms before stimulus onset. Light green: picture naming. Blue: nonspeech environmental sound naming. Magenta: auditory descriptive naming. [0:00-1:15] Horizontal bars indicate the periods in which high-gamma amplitudes were significantly greater during one task than the other based on studentized bootstrap statistics. In the upper row, high-gamma amplitudes during picture naming were contrasted with those during nonspeech environmental sound naming. In the lower row, those during nonspeech environmental sound naming were contrasted with auditory descriptive naming.

[1:16-2:32] Upper horizontal bars indicate the periods in which high-gamma amplitudes were significantly augmented compared to the baseline based on the studentized bootstrap statistics. Lower horizontal bars indicate the periods in which high-gamma amplitudes were significantly attenuated compared to the baseline.

**Video S5. Dynamic connectome matrix.** The color of each box indicates the given naming task(s) that elicited significant direct functional connectivity enhancement between the two corresponding regions of interest (ROIs). Functional connectivity was considered significantly enhanced when two ROIs linked via direct DWI streamlines showed significant (based on the studentized bootstrap statistics), simultaneous, and sustained (≥100 ms) high-gamma co-augmentation. Light green: picture naming. Blue: nonspeech environmental sound naming. Magenta: auditory descriptive naming.

**Video S6. Dynamic change of intra- and inter-hemispheric direct functional connectivity.** Each plot indicates the number of regions of interest (ROI)-pairs showing significantly enhanced direct functional connectivity. Solid line: intra-hemispheric connectivity enhancement. Broken line: inter-hemispheric connectivity enhancement.
