# Supplementary figures and images for: Intra– and inter–hemispheric network dynamics supporting object recognition and speech production
Source: Neuroimage. Author manuscript; Available in PMC 2023 Apr 18. (PMC10112006; doi:10.1016/j.neuroimage.2023.119954)

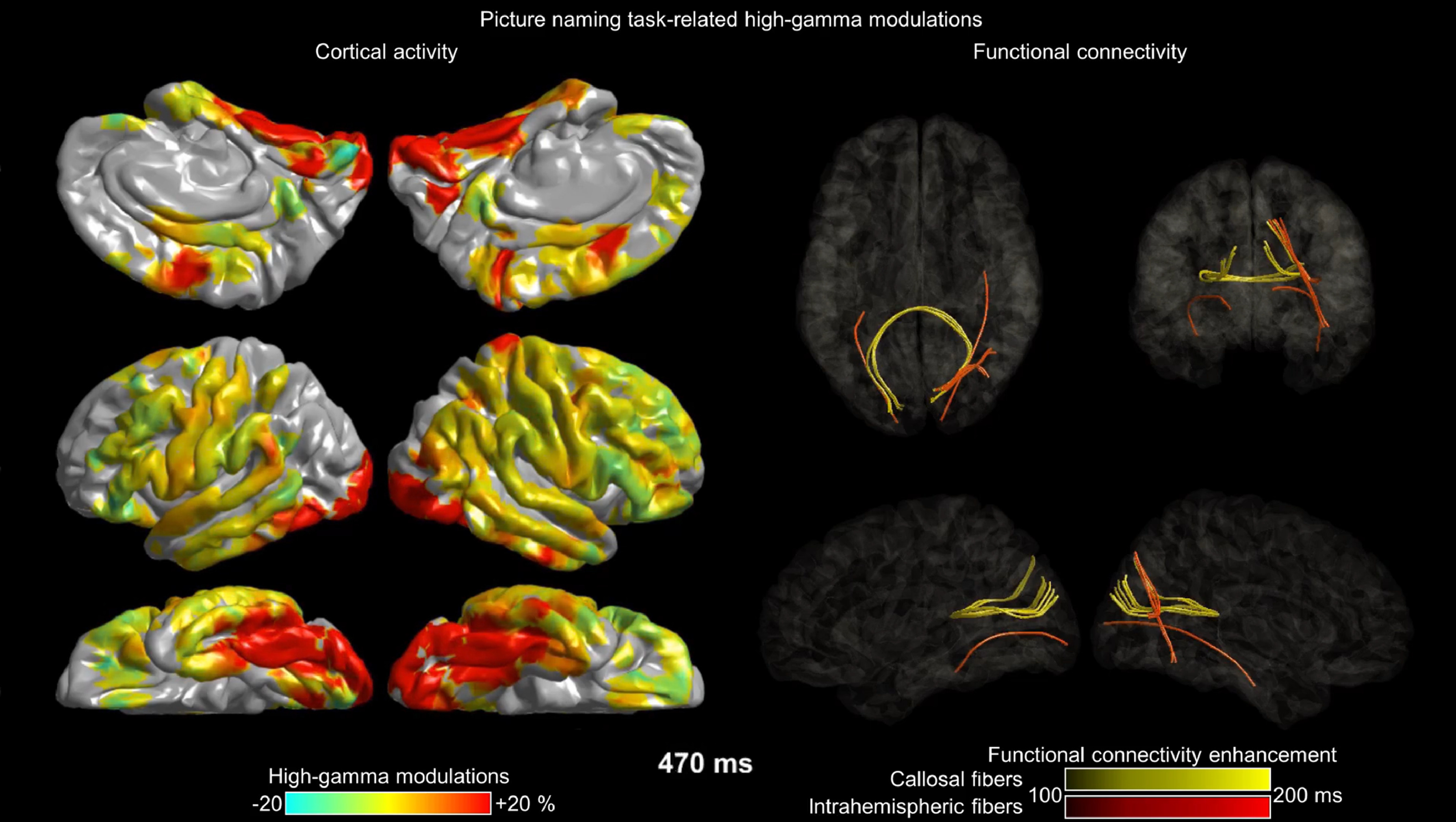

Supplement: 1 [file NIHMS1886331-supplement-1.jpg]

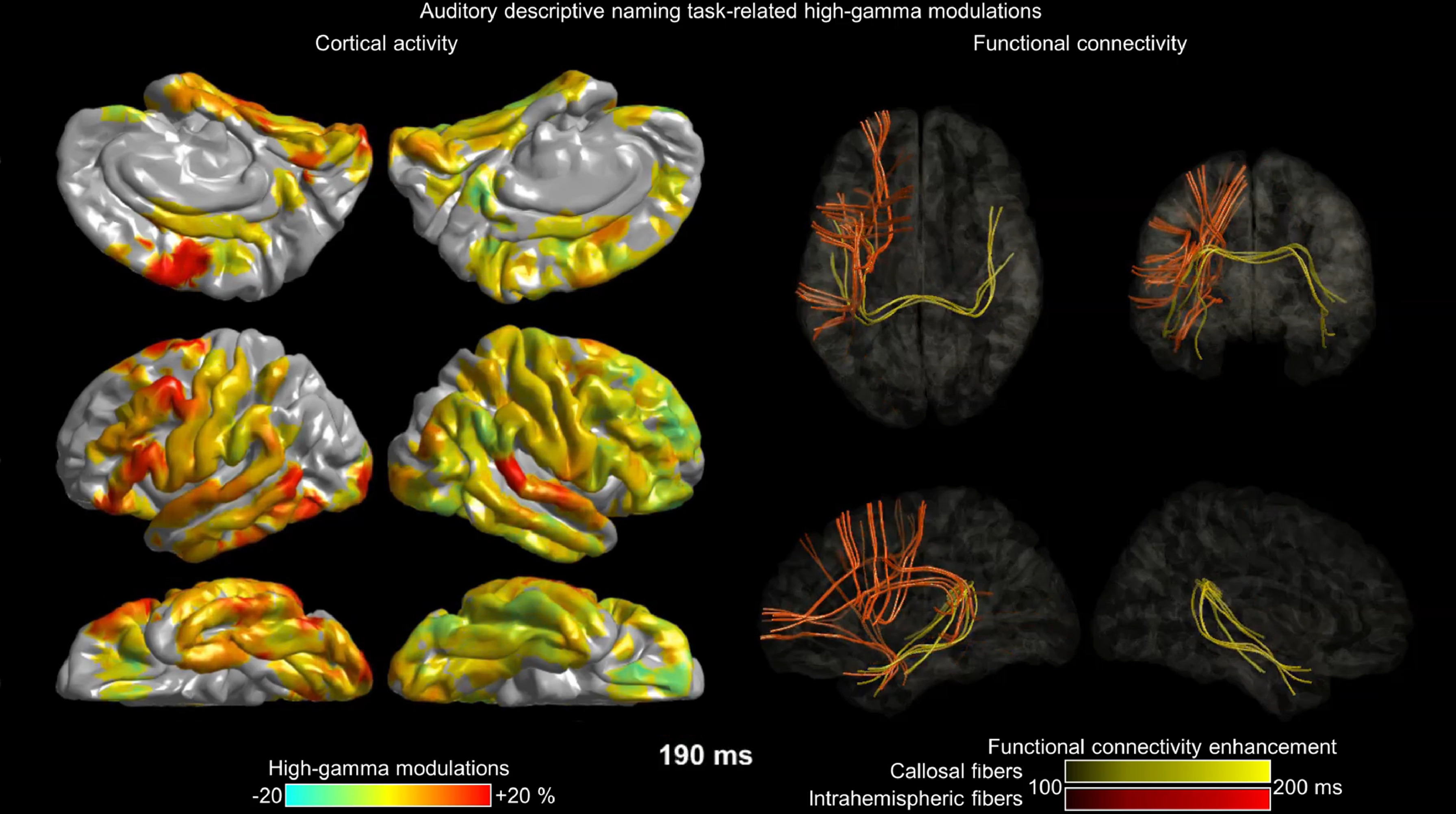

Supplement: 3 [file NIHMS1886331-supplement-3.jpg]

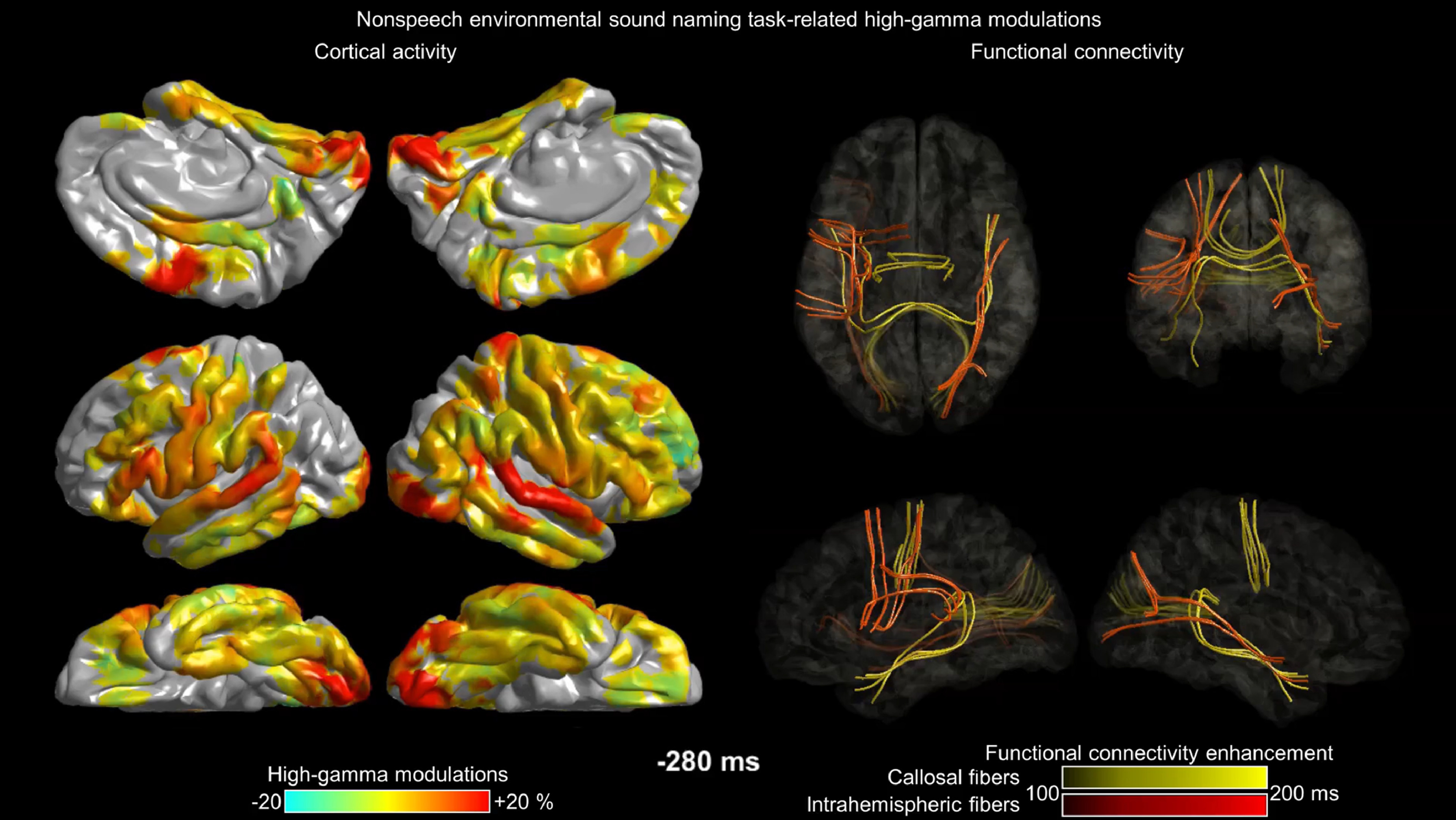

Supplement: 2 [file NIHMS1886331-supplement-2.jpg]

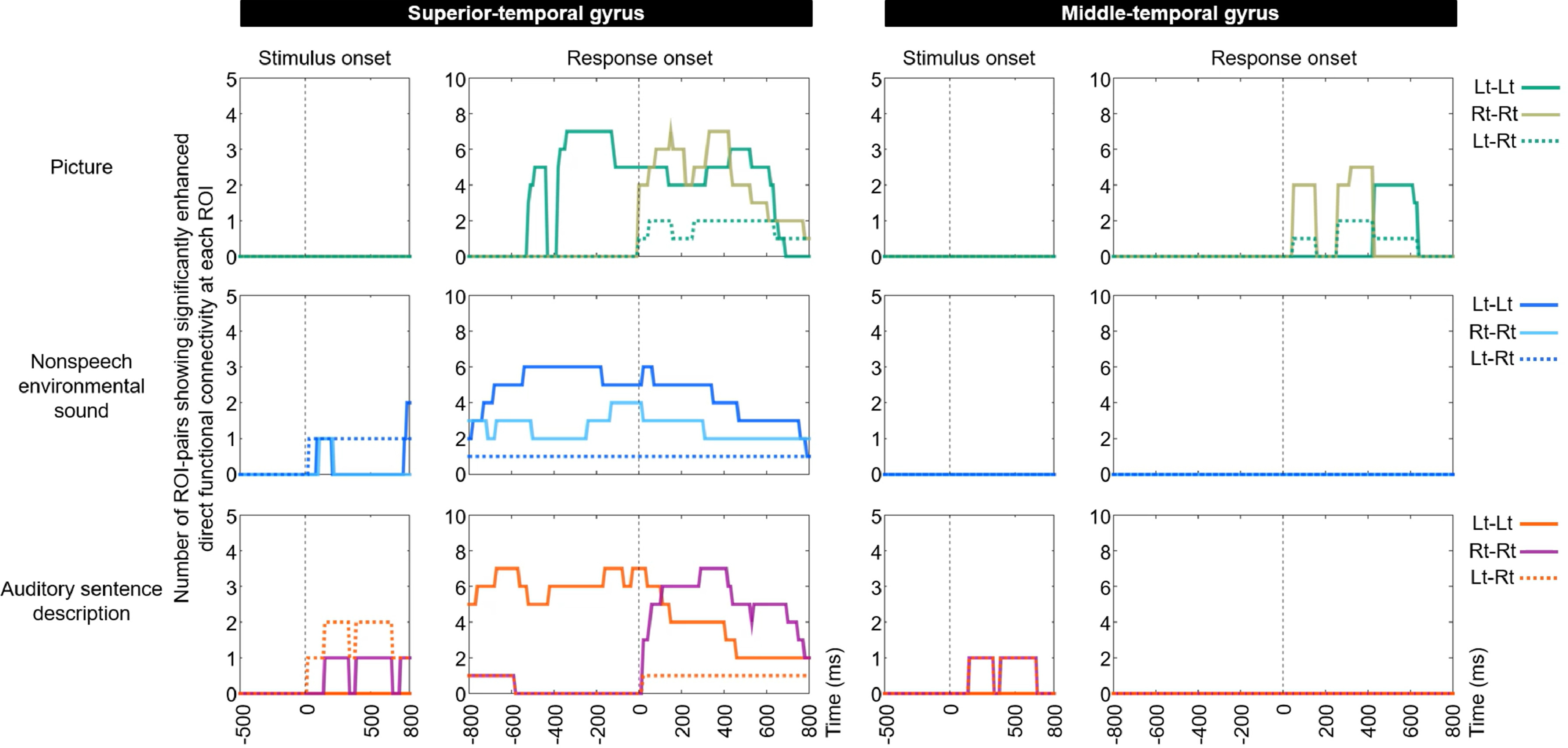

Supplement: 6 [file NIHMS1886331-supplement-6.jpg]

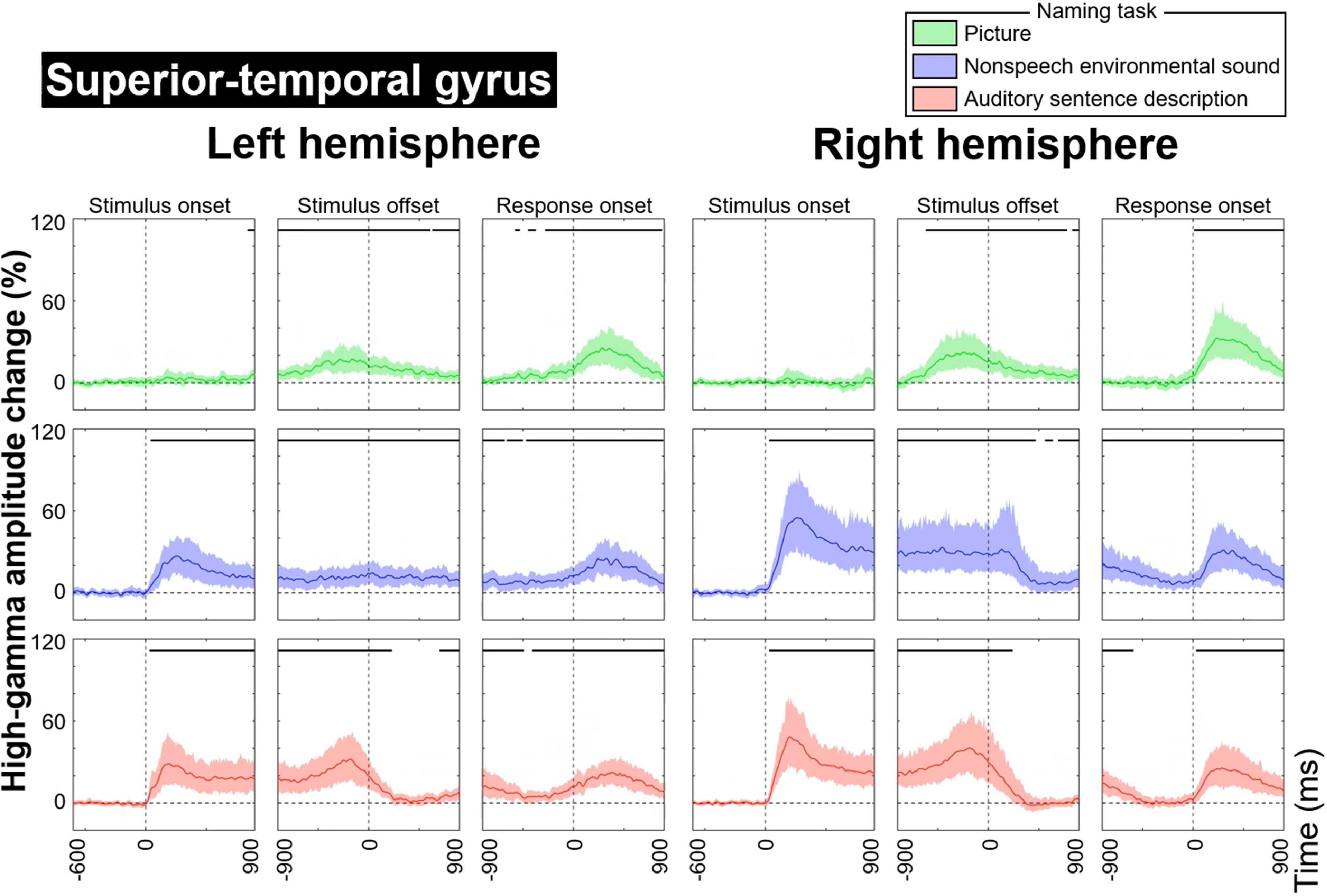

Supplement: 4 [file NIHMS1886331-supplement-4.jpg]

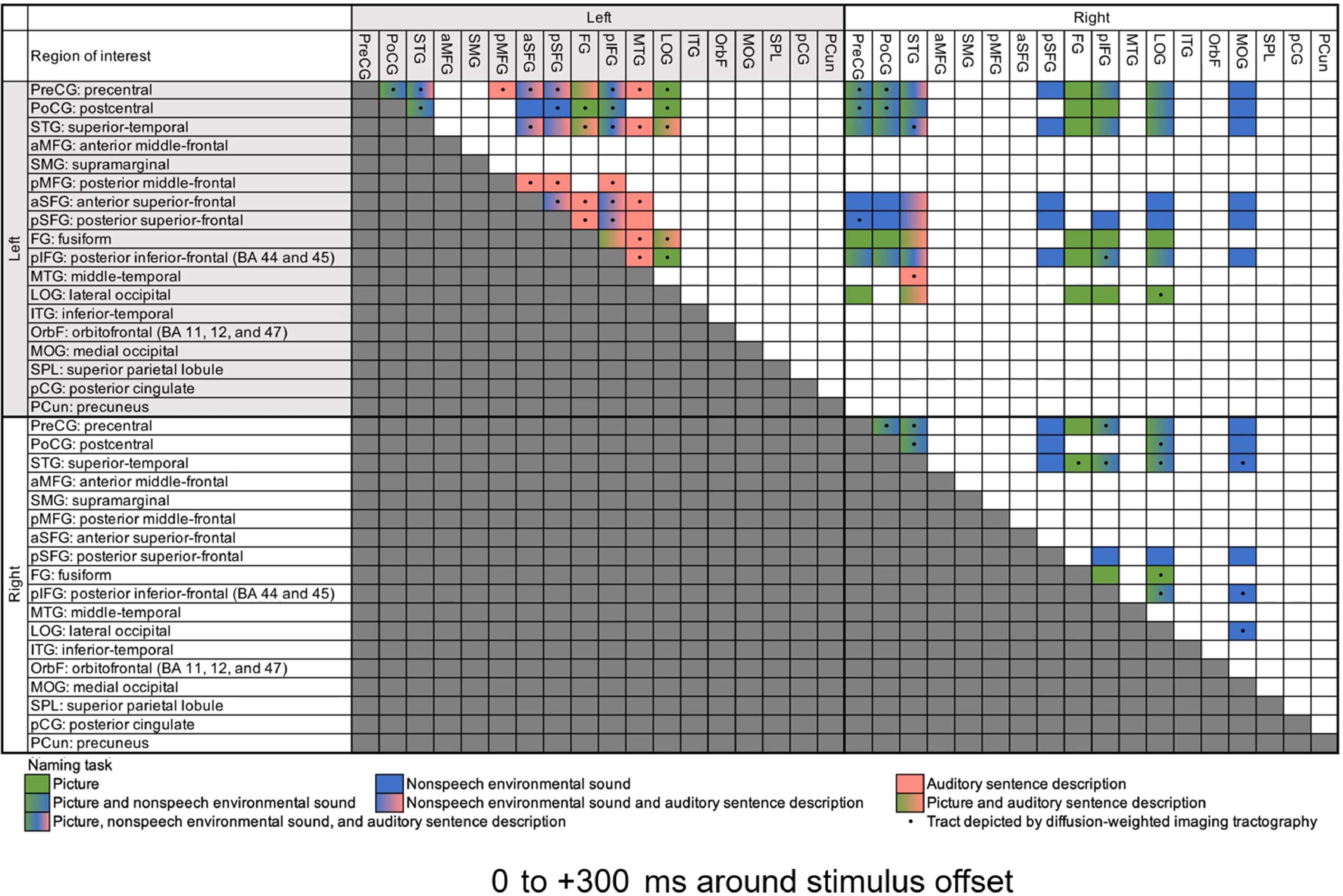

Supplement: 5 [file NIHMS1886331-supplement-5.jpg]
